# Anthocyanins in woody ornamental plants: biosynthesis, regulatory networks, and trade-offs in stress adaptation

**DOI:** 10.3389/fpls.2026.1966821

**Published:** 2026-09-28

**Authors:** Hua Zhang, Yi’na Yao, Mingyang Ni, Yuting Zhang, Chunlong Li, Junhong Zhang, Zaikang Tong

**Affiliations:** 1 Zhejiang Key Laboratory of Forest Genetics and Breeding, Zhejiang A&F University, Hangzhou, China; 2 National Key Laboratory for Germplasm Innovation & Utilization of Horticultural Crops, Huazhong Agricultural University, Wuhan, China

**Keywords:** abiotic stress, anthocyanin, biotic stress, regulatory network, stress tolerance, woody ornamental plants

## Abstract

Anthocyanins are important flavonoid metabolites that are instrumental to plant adaptation to adverse environmental conditions. Increasing evidence has shown that both biotic and abiotic stresses can markedly affect anthocyanin biosynthesis and accumulation, thereby influencing plant defense capacity, stress tolerance, and metabolic reprogramming. In addition to serving as pigments, anthocyanins contribute to plant protection through multiple mechanisms, including reactive oxygen species (ROS) scavenging, metal chelation, photoprotection, and the modulation of stress-related signaling pathways. In ornamental plants, particularly woody ornamentals, anthocyanins not only determine key aesthetic traits such as flower and leaf coloration, but also serve as critical components of the stress defense system. However, compared with herbaceous model plants, the regulatory networks and functional roles of anthocyanins in these species remain insufficiently studied. This review comprehensively explores the molecular regulatory mechanisms of anthocyanin accumulation in woody plants under various stresses. These include biotic stresses (e.g., pathogenic bacteria and insect pests) and abiotic stresses (e.g., drought, salinity, extreme temperatures, heavy metals, and light stress). Furthermore, the dynamic response characteristics of anthocyanins under combined multiple stresses and their potential applications in stress-tolerance breeding are also discussed, providing a theoretical foundation for developing anthocyanin-based strategies to enhance stress tolerance in ornamental horticulture.

## 1 Introduction

Environmental stresses threaten the growth, development, and survival of organisms. In plants, these stresses are categorized as biotic (e.g., pests and pathogens) or abiotic (e.g., temperature extremes, water deficit, salinity, and heavy metal toxicity) (Ma et al., 2020; Shi and Liu, 2021). In response to these stresses, plants exhibit a range of symptoms, such as cellular membrane damage, accumulation of toxic byproducts (e.g., malondialdehyde and hydrogen peroxide), disruption of water balance, and inhibition of photosynthesis and respiration (Zhen et al., 2020; Chauhan et al., 2023; Gowdra et al., 2025). Additionally, nutrient homeostasis and hormone levels are disturbed (Feng et al., 2020; Wang et al., 2025e). To mitigate these adverse effects, plants have evolved adaptive strategies at morphological and physiological levels. These include adjustments to leaf size and orientation, increased nutrient accumulation, enhanced photosynthetic capacity, elevated antioxidant enzyme activity, and the induced synthesis of secondary metabolites (Sharma et al., 2020; Yang et al., 2021; Snoj et al., 2024).

Ornamental plants are cultivated worldwide for their aesthetic and ecological value, and their commercial and ornamental value is largely determined by visual quality traits, including flower color, leaf color, ornamental fruit characteristics, flowering time, and post-harvest longevity (Verdonk et al., 2022). Among the diverse pigments that determine visual quality in ornamentals, anthocyanins are one of the most important and widely distributed, endowing flowers, leaves, and fruits with rich red, purple, and blue hues (Yin et al., 2021; Gao et al., 2024). Unlike crop species, which are bred primary for yield, ornamental plants are particularly susceptible to adverse environmental conditions because their value hinges on these stress-sensitive visual traits (Partap et al., 2023). Abiotic stresses such as drought, extreme temperatures, and water deficit not only impair growth but also severely compromise these aesthetic traits—for instance, by accelerating petal senescence, causing flower color fading or browning, inducing leaf scorch or discoloration, reducing fruit coloration and ornamental quality, shortening flowering period, and decreasing post-harvest longevity (Oren-Shamir and Gal, 2000; Hegde et al., 2020; Toscano et al., 2023; Chachar et al., 2026). However, anthocyanins responses to stress in ornamental plants are governed by a complex regulatory network, and their accumulation can be either suppressed or induced depending on stress intensity and duration. Woody ornamental plants have lifespans ranging from decades to centuries and must endure recurrent, seasonal stresses (e.g., winter cold, summer drought). They have evolved unique “stress memory” mechanisms (Mageroy et al., 2025), and the annual cyclic fluctuations of anthocyanins—for example, red or yellow young leaves in spring in some species, and red, yellow, or purple autumn foliage in others—are a visual manifestation of this long-term adaptive strategy (Verhoeven et al., 2024). In contrast, herbaceous plants or model crops such as *Arabidopsis thaliana*, maize (*Zea mays*), and rice (*Oryza sativa*) exhibit more transient stress response. Moreover, the current understanding of the molecular mechanisms underlying anthocyanin-mediated stress responses is derived largely from herbaceous plants or model crops. Research on woody plants has lagged due to their high genome heterozygosity, difficulty in genetic transformation, and long growth cycles (Maharjan et al., 2025). This knowledge gap is particularly pronounced in woody ornamental plants, where the trade-off between stress adaptation and pigment display (e.g., autumn red leaves or colored bracts) remains understudied, despite their ornamental and landscape importance.

Anthocyanins are naturally occurring polyphenolic pigments widely distributed in plants. They are well known for their strong antioxidant properties, which make them important components of plant defense systems (Saigo et al., 2020). Extensive studies have demonstrated that anthocyanins are closely associated with plant adaptation to both biotic and abiotic stresses. *In vitro* studies have provided quantitative chemical evidence for the radical-scavenging and metal-chelating activities of anthocyanins. For example, in a simulated aqueous environment (pH 7.4), the overall rate constants for anthocyanin-mediated scavenging of hydroperoxyl radicals (HOO•) range from 1.58 × 10^8^ to 7.59 × 10^9^ M^-^¹ s^-^¹, indicating extremely rapid radical scavenging kinetics (Quan et al., 2021). Additionally, the chelation of cyanidin-3-glucoside (C3G) with Fe(III) is pH-dependent, with the strongest binding affinity observed at pH 3.0 (Xie et al., 2018). Although these *in vitro* findings cannot directly prove the functions of anthocyanins in reactive oxygen species (ROS) scavenging and metal chelation within plants, they provide strong chemical support for these proposed *in vivo* functions. The subcellular localization of anthocyanins and ROS determines whether antioxidant actions are feasible *in vivo*. ROS are mainly produced in chloroplasts, mitochondria and the apoplast, while H_2_O_2_ can diffuse across membranes via aquaporins (Zhao et al., 2021). Anthocyanins are synthesized on the endoplasmic reticulum, transiently present in the cytosol, and finally stored in the vacuole (Pucker and Selmar, 2022). Thus, at least two scenarios allow anthocyanins to meet ROS: (i) during their cytosolic/ER residence, they can scavenge ROS generated nearby; (ii) after vacuolar storage, they can neutralize H_2_O_2_ that enters the vacuole via tonoplast aquaporins. Imaging studies confirm that H_2_O_2_ accumulates near anthocyanin-rich vacuoles, and antioxidant efficiency depends on spatial proximity (Gould et al., 2002; Moustaka et al., 2020). Together, these observations explain how vacuolar anthocyanins can effectively participate in ROS scavenging. Complementary *in vivo* evidence comes from physiological studies. Under blue light, leaves rich in anthocyanins exhibit an 18% reduction in the rate coefficient of Photosystem II (PSII) photoinactivation (*k*_i_), demonstrating a significant photoprotective effect (Liu et al., 2023). Therefore, elucidating the regulatory mechanisms of anthocyanin synthesis—particularly the transcriptional, hormonal, and epigenetic networks—and their functional roles under diverse stresses is of fundamental importance for understanding plant adaptation and has practical implications for stress-tolerant crop and forest tree breeding.

This review examines recent advances in our understanding of anthocyanins’ roles in ornamental plant responses to environmental stress. We summarize the biosynthesis, regulatory mechanisms, and functional contributions of anthocyanins in woody ornamental under both biotic and abiotic stresses. We also discuss the key regulatory mechanisms critical for plant adaptation and stress resilience. Furthermore, we explore the dynamic behavior of anthocyanins under stresses combinations and review metabolic engineering strategies aimed at developing anthocyanin-rich plants with enhanced stress tolerance, highlighting their potential for use in stress-resistant breeding programs.

## 2 Regulatory mechanism of anthocyanin synthesis

Anthocyanins are flavonoids that contain two benzene rings and one heterocycle, which confer diverse biological activities (Strack et al., 1989). Their biosynthesis originates from the phenylpropanoid pathway (**Figure 1**). The phenylpropanoid pathway begins with phenylalanine, which is converted to trans-cinnamic acid by phenylalanine ammonia-lyase (PAL). This intermediate is subsequently channeled into the flavonoid pathway via subsequent enzymatic reactions. Ultimately, anthocyanins are synthesized and transported to vacuoles. Specifically, phenylalanine is first converted to 4-coumaroyl-CoA via enzymatic reactions. Then, 4-coumaroyl-CoA and malonyl-CoA are converted by a series of enzymes to form dihydroflavonol. These enzymes include chalcone synthase (CHS), chalcone isomerase (CHI), and flavanone-3-hydroxylase (F3H). Finally, colorless dihydroflavonol is transformed into various colored anthocyanins by dihydroflavonol-4-reductase (DFR), anthocyanidin synthase (ANS), and UDP-glucose: flavonoid 3-O-glucosyltransferase (UFGT) (Yang et al., 2024b; Huang et al., 2025; Zhang et al., 2025b). The biosynthesis of anthocyanins is precisely controlled by multilayer regulatory networks, including transcriptional regulation, epigenetic modifications, hormone signal transduction, and transport/vacuolar compartmentation. These mechanisms work synergistically to help plants adapt to various environmental stresses (e.g., drought, low temperature, UV radiation). **Supplementary Table S1** summarizes the key regulatory genes, their functional categories, anthocyanin content changes, species information, and corresponding environmental factors cited in this review.

**Figure 1:**
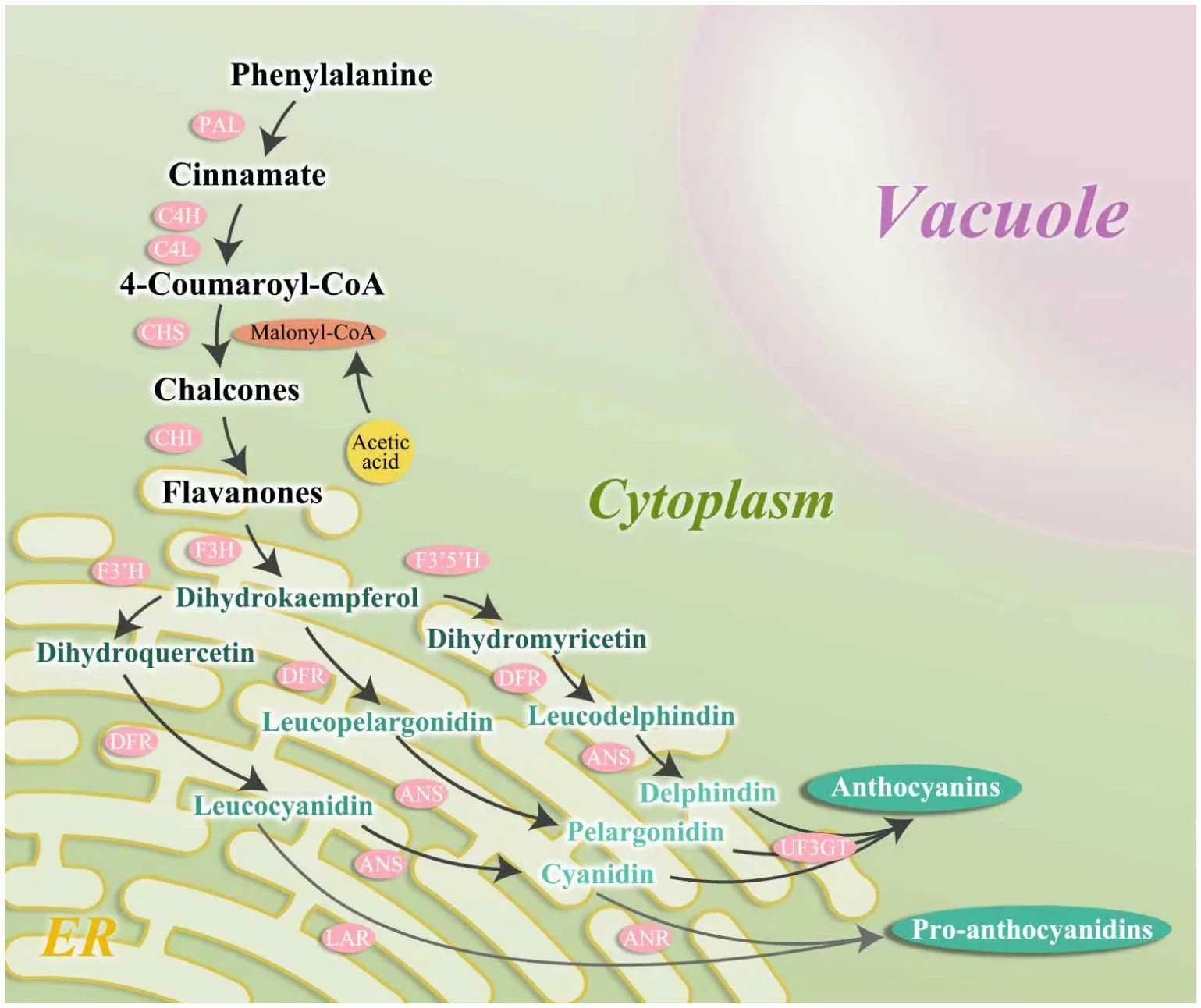
Schematic illustration of the anthocyanin biosynthesis pathway. PAL, phenylalaninammonia-lyase; C4H, cinnamate 4-hydroxylase; 4CL, 4-Coumarate, Coenzyme A Ligase; CHS, chalcone synthase; CHI, chalcone isomerase; F3H, flavanone 3-hydroxylase; F3′H, flavanone 3′-hydroxylase; F3′5′H, flavanone 3′5′-hydroxylase; DFR, dihydroflavonol 4-reductase; ANS, anthocyanidin synthase; UF3GT, UDP-glucose:flavonoid 3-O-glucosyltransferase; LAR, leucoanthocyanin reductase; ANR, anthocyanin reductase.

### 2.1 Transcriptional regulation

Anthocyanin biosynthesis involves not only structural genes but also the precise regulatory control of transcription factors (TFs). MYB (Myeloblastosis), bHLH (basic Helix-Loop-Helix), and WD40 (Tryptophan-Aspartic acid 40 repeat) proteins are well-characterized TFs that interact to form the MBW complex, which activates the expression of structural genes and regulates anthocyanin biosynthesis (Cheng et al., 2024). Specific members of other TF families (Xu et al., 2023a; Lu et al., 2025), including WRKY (named after the conserved WRKYGQK motif), NAC (NAM, ATAF1/2, and CUC2), bZIP (basic leucine zipper), and ERF (Ethylene Responsive Factor), also bind to the promoters of structural genes and modulate their expression. These TFs can function as either activators or repressors. For example, in apple (*Malus domestica*) fruit exposed to light after bag removal, the ERF TF MdERF109 is induced and directly activates the transcription of *MdCHS*, *MdUFGT*, and *MdbHLH3*, thereby promoting light-induced anthocyanin biosynthesis (Ma et al., 2021). Similarly, *StWRKY13* activates *StCHS*, *StF3H*, *StDFR*, and *StANS* in potato (*Solanum tuberosum*) tubers, leading to anthocyanin accumulation (Zhang et al., 2022a). In contrast, the MdNAC14-like protein represses anthocyanin synthesis by binding to the promoters of *MdMYB9*, *MdMYB10*, and *MdUFGT* (Xu et al., 2023a). Additionally, the bZIP TF SlAREB1 directly interacts with the promoters of *SlDFR* and *SlF3’5’H*, two structural genes, and regulates anthocyanin biosynthesis in tomato (*Solanum lycopersicum*) seedlings at low temperatures via an ABA-dependent pathway (Xu et al., 2023b). Environmental stresses modulate the expression or activity of these transcription factors through upstream signaling molecules, thereby determining the final level of anthocyanin accumulation.

### 2.2 Epigenetic regulation

Epigenetic regulation of anthocyanin metabolism involves multiple reversible modifications, primarily DNA methylation and histone modifications (**Figure 2**). Unlike genetic mutations, these modifications induce heritable changes in gene expression and phenotype without altering the genomic DNA sequence (Sun et al., 2023). DNA methylation, catalyzed by DNA methyltransferases, generally represses transcription when it occurs in promoter regions of structural or regulatory genes (Li et al., 2022). For instance, hypermethylation of anthocyanin structural gene promoters in apple directly suppresses their transcription, which correlates with a color shift from red to green (Xu et al., 2024). Histone modifications—including acetylation, methylation, phosphorylation, and ubiquitination—occur at specific amino acid residues in the N-terminal tails of histones and modulate gene expression by altering chromatin accessibility (Chachar et al., 2022). These modifications can either activate or repress anthocyanin regulation. In poplar (*Populus* spp.), knockout or inhibition of the histone demethylase gene *PtrJMJ25* relieves transcriptional repression, thereby promoting anthocyanin accumulation (Fan et al., 2018). By contrast, in grape (*Vitis vinifera*), the histone deacetylase *VvHDAC19* represses target gene expression through histone deacetylation, reducing anthocyanin accumulation (Jia et al., 2023).

**Figure 2:**
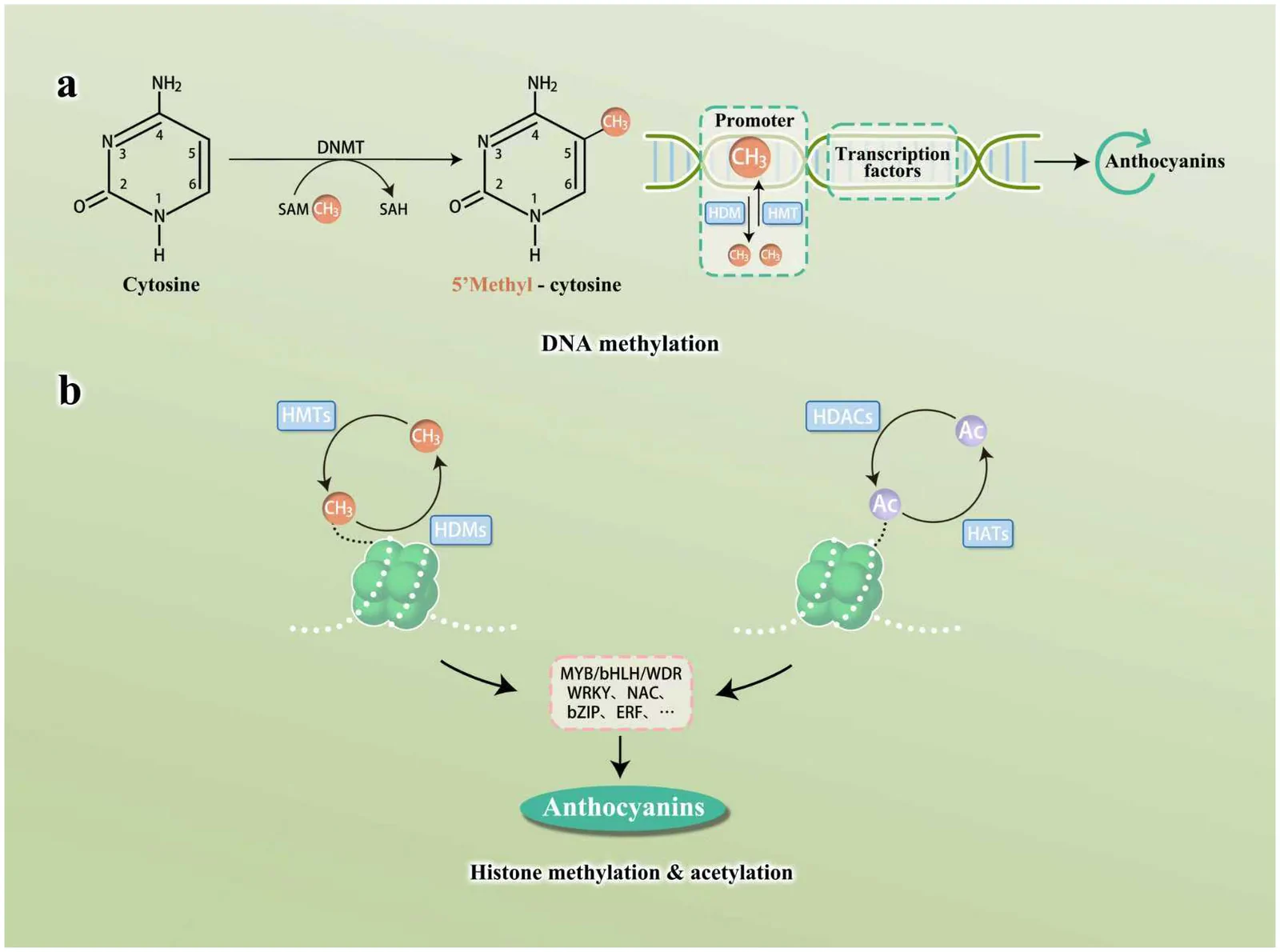
Schematic illustrating the epigenetic regulatory mechanisms governing anthocyanin biosynthesis. It categorized into two modules: DNA methylation **(a)**, histone methylation/acetylation **(b)**.

Despite these advances, the epigenetic regulatory landscape of anthocyanins in woody plants remains poorly characterized compared with herbaceous model systems such as *Arabidopsis* and maize, where comprehensive and refined regulatory networks have been established (Erhard et al., 2013; Zheng et al., 2019). This knowledge gap is largely attributable to technical barriers that are particularly pronounced in woody plants (Maharjan et al., 2025): the lack of publicly available reference epigenomes and the high cost of high-throughput epigenomic assays have limited large-scale comparative studies, whereas the low efficiency of stable genetic transformation impedes validating how epigenetic marks causally function. The long generation times of these species further preclude the rapid generation of mutants that would allow functional epigenetics to move beyond correlation. In the context of anthocyanin regulation, this transformation bottleneck is particularly detrimental, as it precludes the functional dissection of methylation marks at specific biosynthetic loci—a routine practice in herbaceous models. Consequently, more than half of the published studies in woody plants remain correlative, relying on associations between methylation levels or histone modification states and anthocyanin content, without causal validation through genetic or epigenetic perturbation (Gu et al., 2024).

A particularly intriguing observation comes from *Citrus sinensis*, where fungal infection induces both elevated expression of anthocyanin biosynthetic genes (*DFR* and *RUBY*) and increased DNA methylation levels in their promoter regions (Sicilia et al., 2021). This seemingly paradoxical co-occurrence challenges the simplistic view that promoter methylation invariably suppresses gene expression. Drawing an analogy to the rice blast resistance gene *Pib*, high levels of CG methylation at the promoter do not preclude induction upon fungal infection (Sicilia et al., 2021), suggesting that methylation may play a positive or modulatory role in specific biotic contexts. They further acknowledged that the McrBC-based assay employed in their study may lack single-base resolution, raising the possibility that critical TF-binding motifs are hypomethylated, even though the broader promoter region is hypermethylated. They also raised the possibility that post-transcriptional mechanisms—such as RNA-binding proteins that modulate mRNA stability or processing—may contribute to the elevated transcript levels observed in inoculated fruits. Finally, the progressive demethylation observed in control fruits over time, as opposed to the sustained methylation in infected ones, suggests that infection may actively maintain a methylation configuration that, rather than repressing, permits the induction of defense-associated anthocyanin synthesis. Collectively, these considerations underscore that the functional outcome of DNA methylation is highly context-dependent and cannot be generalized from the paradigm established in herbaceous models.

The blood orange example illustrates that the functional outcome of promoter methylation is not monolithic but rather depends on the specific regulatory context—including the nature of the stress, the availability of transcriptional activators, and the developmental stage of the tissue. Unravelling such context-dependent regulatory logic in woody plants demands a deliberate shift from correlative descriptions to mechanistic dissection. In practice, this shift can be accelerated by concentrating initial epigenomic resource-building efforts on a few genetically tractable woody representatives—such as *Populus* and *Citrus*, for which high-quality reference genomes and efficient transient transformation systems are already available—rather than dispersing efforts across a wide array of ornamental species. Transient assays, in particular, can serve as a rapid functional screening platform to test the causal effects of specific methylation or histone marks before committing to the time-consuming generation of stable lines. Ultimately, the development of programmable epigenome-editing tools—applied to these validated loci—is essential for moving beyond correlative associations. By strategically integrating these complementary approaches, we can progressively understand anthocyanin regulation in woody ornamentals at a mechanistic depth that has long been achieved in herbaceous models.

### 2.3 Hormonal signaling regulation

Hormonal signaling networks serve as a key hub integrating environmental stress signals with the reprogramming of secondary metabolism. Multiple hormones, through synergistic or antagonistic interactions, regulate the activity of the MBW transcriptional complex, thereby controlling the expression of structural genes in the anthocyanin biosynthetic pathway (**Figure 3**). Abscisic acid (ABA), as a core stress hormone, promotes the expression of anthocyanin structural genes under drought, salt, and cold stresses by upregulating MYB/bHLH transcription factors (e.g., the MYB1-bHLH3 complex) (An et al., 2021). Ethylene, via its downstream ERF transcription factors, directly or indirectly upregulates the expression of structural genes such as *DFR*, *ANS*, and *UFGT*, thereby inducing anthocyanin accumulation under drought stress (Chang et al., 2023). Jasmonic acid (JA) activates the MBW complex by upregulating its core components via key transcription factors in its signaling pathway (e.g., MYC2), thereby promoting anthocyanin synthesis and enhancing plant defense responses (An et al., 2016).

**Figure 3:**
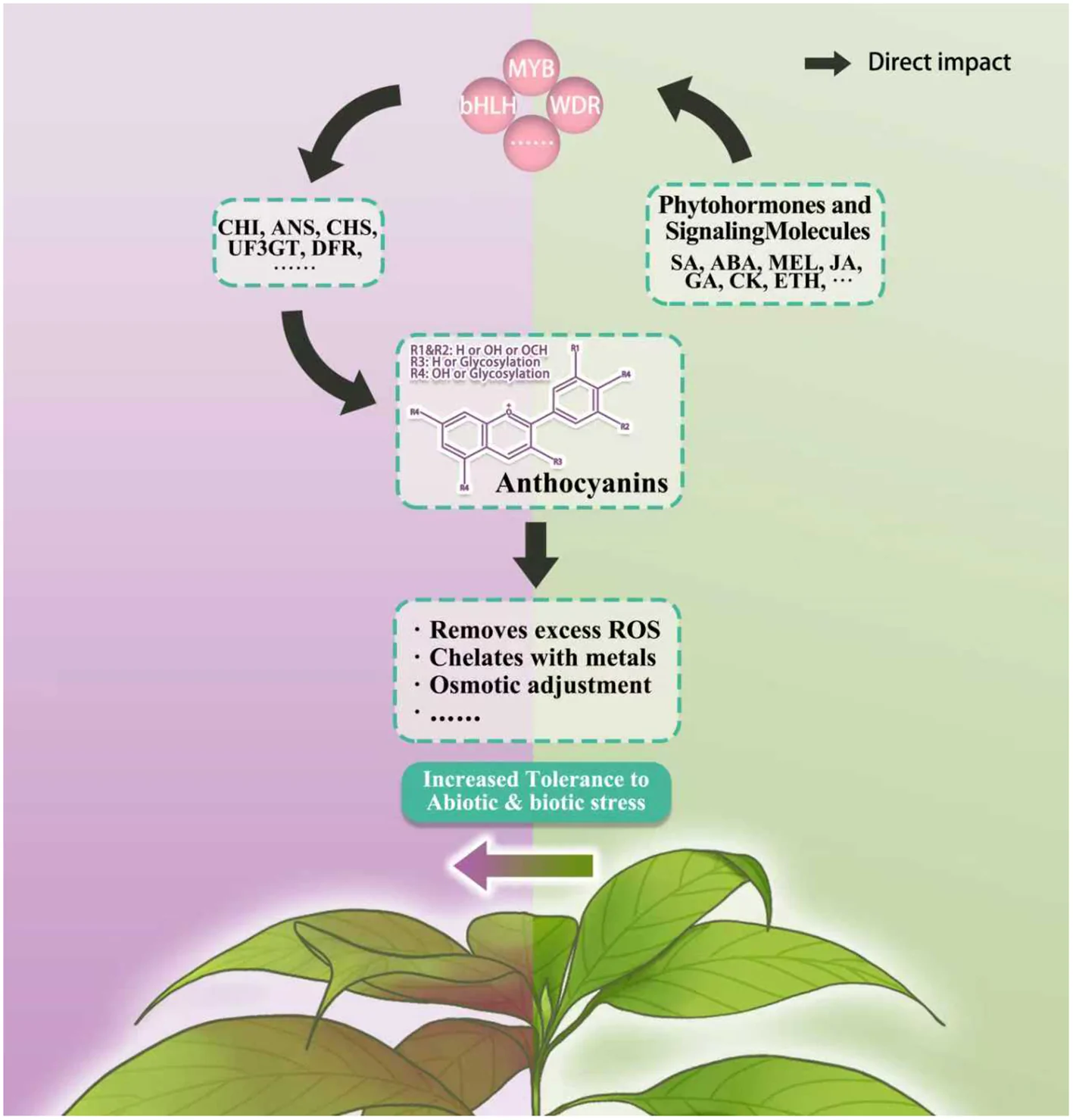
Hormonal signaling networks regulating anthocyanin biosynthesis under stresses.

Although these hormones converge on the MBW complex, their integration under combined stresses is not simply additive. At the molecular level, this non-additive integration is achieved through at least three mechanisms: (1) Synergistic amplification—the ethylene-responsive transcription factor *VvERF045* directly activates both anthocyanin structural genes (*VvUFGT*, *VvDFR*) and the biosynthetic genes for ABA and BR. Critically, *VvERF045* forms a heterodimer with *VvMYB62*, synergistically enhancing the transcriptional activation of the aforementioned target genes, thereby integrating three hormonal pathways through a single regulatory factor to produce a supra-additive effect (Li et al., 2026b); (2) Antagonistic—auxin suppresses anthocyanin biosynthesis through the AUX/IAA-ARF signaling module, whereas ethylene counteracts this effect via ERF transcription factors, with the final phenotype depending on the relative strength of the two hormonal signals (Li et al., 2023); (3) Signal prioritization—jasmonic acid (JA) promotes anthocyanin accumulation by inducing JAZ protein degradation, thereby relieving the transcriptional repression imposed by JAZ proteins on MYB and bHLH subunits within the MBW complex. In contrast, ethylene, via EIN3/EIL1 transcription factors that coordinate with JAZ proteins, directly binds and inhibits the activity of bHLH-MYB subunits, negatively regulating anthocyanin accumulation. The two pathways achieve signal crosstalk through competitive binding to JAZ and the MBW complex, with JA signaling dominating the regulatory hierarchy (Song et al., 2022).

Conceptually, this integration can be viewed as a three-layer decision framework: at the input layer, stress signals are encoded by hormone-specific signatures (ABA for osmotic stress, ethylene for mechanical/cold stress, JA for wounding); at the integration layer, the MBW complex serves as a computational node where converging signals are weighted through synergistic amplification or antagonistic competition—with hierarchical dominance (e.g., JA overriding ethylene signals) determined by the relative strength and timing of each hormonal input; and at the output layer, the integrated signal is translated into a graded transcriptional response that adjusts anthocyanin accumulation according to the perceived stress landscape. Collectively, these mechanisms position the MBW complex as a molecular decision-hub that computes a context-appropriate anthocyanin output based on signal intensity, timing, and tissue context. Future studies should move beyond single-hormone analyses to combinatorial manipulations (e.g., multi-hormone dose–response experiments and sequential stress treatments), which are essential for validating this integration framework and for designing precise interventions to optimize anthocyanin accumulation in woody ornamentals under complex stress scenarios.

### 2.4 Transport and vacuolar sequestration

For anthocyanins to fulfill their biological functions, efficient transport and vacuolar storage are essential. Newly synthesized anthocyanins are generated in the cytoplasm or on the surface of the endoplasmic reticulum and must then be transported into the vacuole for storage via glutathione S-transferases (GSTs) and membrane transporters (e.g., multidrug and toxic compound extrusion (MATE) and ATP-binding cassette (ABC) transporters) (Zhao, 2015; Pucker and Selmar, 2022) (**Figure 4**); the acidic environment of the vacuole ensures the chemical stability of anthocyanins by maintaining their flavylium cation form. Under stress conditions, changes in transport efficiency directly govern the rate of anthocyanins accumulation and their subcellular distribution, thereby affecting their antioxidant capacity to scavenge ROS.

**Figure 4:**
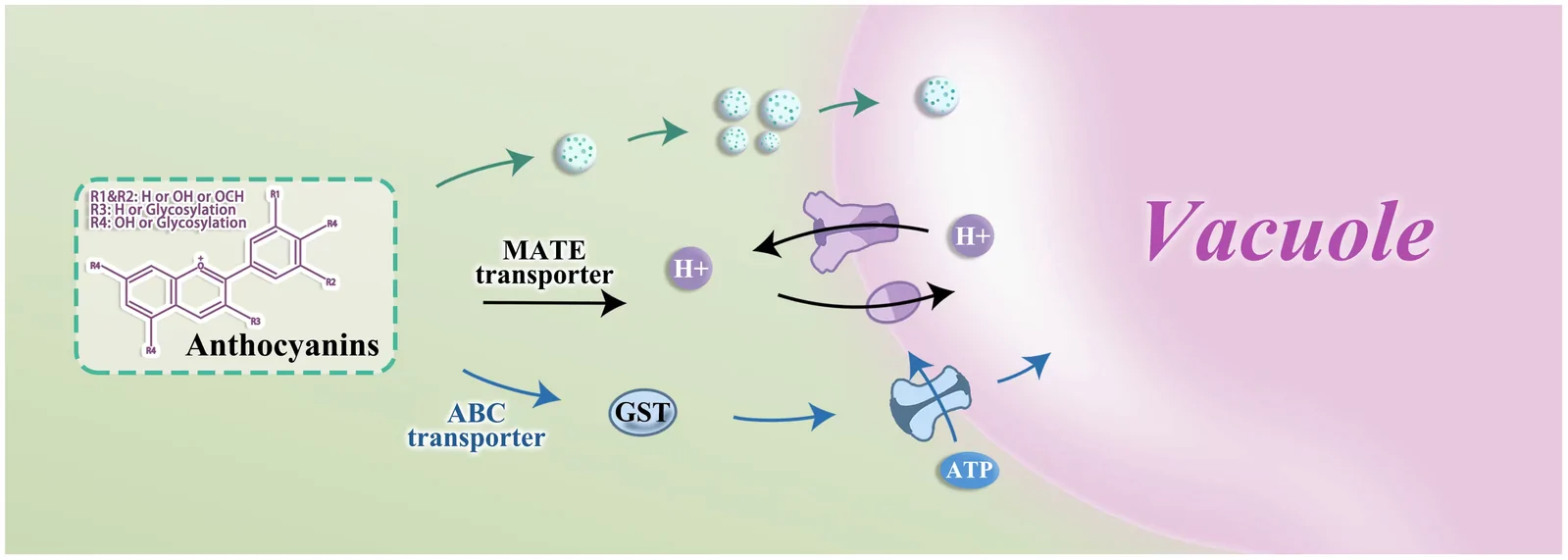
Schematic illustration of the anthocyanin vacuolar trafficking.

A recent finding challenges the traditional view of GSTs merely as passive transporters. It was demonstrated *in vitro* that that certain anthocyanin-related GSTs can catalyze the dehydration of leucoanthocyanidin dioxygenase (LDOX) products to form anthocyanins (Eichenberger et al., 2023). This suggests that GSTs may play a dual role—not only transporting pigments to the vacuole but also participating in the final biosynthetic step. If this catalytic activity is conserved in *planta*, the current linear pathway boundary between synthesis and transport would no longer apply, allowing synthesis and transport to be spatiotemporally coupled at the level of GSTs. The major implication of this coupling is that the transport step is no longer a passive post-synthesis event but can be coordinated with the terminal synthesis rate, thereby channeling unstable anthocyanin precursors directly into the vacuole while minimizing their retention and cytosolic degradation.

From the perspective of transport regulation, the dual function of GSTs implies that changes in their expression levels will affect the yield of terminal products and the amount of substrate available for transport. Therefore, transport efficiency is not governed solely by the transporter activity, but increasingly recognized as being coupled to the catalytic efficiency of GSTs. When responding to stress, this coupling enables plants to optimize both anthocyanin synthesis flux and vacuolar sequestration rate through the regulation of a single GST node, without the need to separately regulate synthesis enzymes and transporters. Future studies should focus on whether this catalytic activity is conserved in woody plants, and whether there is physical or functional coupling between the catalytic and transport functions of GSTs at the subcellular level (e.g., at the ER–vacuole interface). If confirmed, the relative contribution of this coupling to the overall transport flux would need to be further quantified to assess whether it reshapes our understanding of the initial steps of anthocyanin transport.

## 3 Anthocyanin synthesis and potential roles under biotic stresses

### 3.1 Pathogen attacks

Pathogen-induced losses in horticultural crops account for approximately 20–30% of global production annually, and up to 50% in hot and humid regions (Terry et al., 2011). To counter these threats, woody ornamentals often accumulate anthocyanins at infection sites. Integrating available evidence, we propose a heuristic working model: pathogen perception triggers a hormone surge (salicylic acid (SA), jasmonic acid (JA), and ethylene), which converge to trigger transcriptional reprogramming—primarily via the MBW complex and, in certain lineages, alternative modules such as the NPR1-TGA axis—to upregulate structural genes (e.g., *CHS*, *DFR*, *ANS*, *FLS*), resulting in localized anthocyanin deposition that may contribute to antimicrobial defense.

However, this model is largely extrapolated from a single complete validation in apple *(Malus × domestica)* and, as detailed below, its universality across woody angiosperms remains unproven. At the hormone level, SA, JA and ET are consistently induced upon infection, yet their relative dominance varies. In apple, SA accumulation is prominent during *Valsa mali* and *Botryosphaeria dothidea* attack, and has been functionally linked to the *MdNPR1*-*MdTGA2.2* module (Li et al., 2024b). In contrast, *Chrysanthemum*–*Botrytis cinerea* interactions show a more balanced involvement of JA and ET, with responsiveness that correlates with cultivar-specific pigmentation (Suraj et al., 2025, 2026). Blood orange, while displaying elevated *DFR/RUBY* expression upon fungal challenge, also exhibits increased promoter DNA methylation, suggesting an epigenetic gating of hormone-driven transcription not observed in apple or chrysanthemum (Sicilia et al., 2021).

At the transcriptional level, while the MBW complex is widely accepted as a terminal activator, recent evidence implicates additional regulators. WRKY transcription factors have been shown to mediate flavonoid pathway activation upon pathogen infection in woody roots (e.g., *Populus*) (Li et al., 2024a), while BBX-family proteins coordinate anthocyanin biosynthesis and ROS homeostasis during infection, acting either upstream of or in parallel with MBW (Zhang et al., 2022b; Wang et al., 2025b). Apple offers the most complete validation of the NPR1-TGA route, but direct evidence for this module outside *Malus* is scant. Intriguingly, long-term breeding for ornamental traits in chrysanthemum appears to have attenuated the inducibility of this transcriptional cascade in certain cultivars, revealing a genetic trade-off between constitutive pigmentation and pathogen-triggered activation (Suraj et al., 2026).

Functionally, accumulated anthocyanins act as a chemical barrier: exogenous anthocyanin application reduces susceptibility to *Penicillium digitatum* in citrus (Lin et al., 2021), and *in vitro* extracts from *Clitoria ternatea* and *Dioscorea alata* show antimicrobial activity (Mahmad et al., 2018). Nevertheless, a critical caveat must be raised: anthocyanins are co-induced with a cocktail of other defensive metabolites, including condensed tannins (proanthocyanidins), phenolics, and phytoalexins. In woody perennials such as poplar, proanthocyanidins share the same upstream flavonoid pathway and have been independently implicated in pathogen resistance (Lan et al., 2026). Consequently, the relative contribution of anthocyanins per se versus these co-induced compounds has not been quantitatively resolved in any woody ornamental species. Moreover, loss-of-function studies directly silencing anthocyanin biosynthesis (e.g., *ANS* or *UFGT*) in woody tissues under pathogenic challenge remain absent, meaning the *in vivo* necessity of anthocyanins for resistance is still a matter of correlation, not causation. Epigenetic modifications add further complexity but appear context-dependent; DNA hypermethylation in blood orange does not abolish *DFR* upregulation, suggesting that other regulatory layers (e.g., hormone-activated WRKY/BBX modules) can override epigenetic marks, potentially serving as a fine-tuning mechanism for response amplitude (Sicilia et al., 2021).

In summary, the core framework—pathogen perception → SA/JA/ET → MBW/NPR1-TGA → structural genes → anthocyanins → antimicrobial activity—is supported by correlative evidence across multiple woody taxa, with apple providing the only complete pathway validation to date. **Table 1** summarizes the cross-species evidence, distinguishes established facts from extrapolations, and highlights unverified steps for each node. The MBW complex emerges as the most conserved transcriptional core, while WRKY and BBX transcription factors represent newly recognized regulatory nodes that may operate upstream of or in parallel to MBW during pathogen defense. However, three critical caveats must be addressed before this model can be accepted as universally applicable. First, the *in vivo* necessity of anthocyanins for pathogen resistance has not been established in any woody ornamental, as loss-of-function studies in woody tissues under pathogenic challenge are absent. Second, the functional contribution of anthocyanins cannot be disentangled from co-induced metabolites—particularly proanthocyanidins, which share the same upstream flavonoid pathway and are independently implicated in defense—without targeted metabolomic fractionation or genetic dissection. Third, the NPR1-TGA module, while elegantly validated in apple, has not been demonstrated outside Malus, and the role of epigenetic modifications remains correlative rather than mechanistically defined. Addressing these gaps will require moving beyond heterologous overexpression and phenotypic correlation toward direct causality through gene editing in representative woody species, combined with time-resolved multi-omics to resolve the temporal order of signaling events and defensive outputs.

**Table 1:** Cross-species comparison of pathogen-induced anthocyanin accumulation by mechanistic node.

Species	Hormone signaling	Transcriptional reprogramming	Structural gene activation	Antimicrobial function & phenotype	Epigenetic regulation	Evidence level
Apple (*Valsa mali*/*Botryosphaeria dothidea*)	SA dominant; linked to *MdNPR1*	MBW and NPR1-TGA (validated)	*CHS*, *DFR*, *ANS*, *FLS* induced	Red pigmented rings; no KO tested	NR	Validated pathway
*Chrysanthemum* (*Botrytis cinerea*)	JA/ET prominent; cultivar-dependent	MBW induced; breeding-attenuated	*CHS*, *DFR* induced	Petal accumulation; no mutant	NR	Correlative
Blood orange (fungal infection)	SA/JA and DNA methylation	DFR/RUBY induced despite hypermethylation	*DFR* induced	Not tested *in vivo*	Promoter hypermethylation; does not block transcription	Correlative
Poplar (*Populus* spp./canker disease)	SA/JA predicted (transcriptomic)	WRKY/BBX modules implicated (RNAseq)	*CHS*, *F3H* upregulated (RNAseq)	Induced in infected roots; no functional assay	NR	Correlative
Conserved core & Key gaps	SA/JA/ET universally induced; dominance varies; WRKY/BBX emerging as additional nodes	MBW is core; NPR1-TGA is apple-validated module; no validation outside Malus	*CHS* & *DFR* are the most conserved markers	*In vitro* activity confirmed; *in vivo* necessity untested in all species; contribution vs. PAs/other metabolites unquantified	Epigenetic marks present functional link to signaling unknown	Framework remains heuristic

### 3.2 Insect pests

While anthocyanin-mediated insect resistance has been experimentally validated in herbaceous models (e.g., *Petunia hybrida* purified compound assays) (Johnson et al., 2008), its relevance to long-lived woody ornamentals remains uncertain. Extrapolating from annual herbs risks overlooking the unique ecological and physiological context of perennials. Rather than treating this as an unexplored completely open question, we propose three testable, competing hypotheses to reframe the enquiry:

Hypothesis 1 (Visual Deterrence Hypothesis): In large woody plants, anthocyanins primarily act as visual signals that alter herbivore host selection (oviposition/feeding preference) through dark pigmentation, rather than through post-ingestion toxicity.

Hypothesis 2 (Metabolic By-product Hypothesis): Anthocyanin accumulation under herbivory is a non-adaptive by-product of stress-induced metabolic reprogramming (e.g., carbon reallocation to wounded sites, or ROS scavenging at feeding lesions), with no selected defensive function per se.

Hypothesis 3 (Direct Toxicity Hypothesis): The core hormone (JA/ET) → MBW → anthocyanin cascade directly produces toxic or feeding-deterrent compounds, similar to the herbaceous model, but potentially modulated by long-distance stress memory.

Empirical evidence from woody perennials can be parsed through these lenses. The strongest support for H1 comes from transgenic citrus overexpressing anthocyanin regulators: increased leaf pigmentation significantly reduced host preference and survival of the Asian citrus psyllid (*Diaphorina citri*), with behavioral assays confirming visual deterrence as a major component (Killiny et al., 2025). Ecologically, in *Eucalyptus* species, red/purple juvenile leaves—rich in anthocyanins—correlate with reduced psyllid infestation, with field experiments suggesting that visual contrast against green foliage affects host-finding behavior (Farnier and Steinbauer, 2016). Similarly, in the New Zealand *Horopito* (*Pseudowintera colorata*), red leaf margins have been shown to function as a visual signal of chemical defence, with wider red margins containing higher concentrations of both anthocyanins and the defensive compound polygodial, and incurring less natural herbivory (Cooney et al., 2012). These observations collectively support H1 and, to a lesser extent, H3.

Evidence for H2 is circumstantial but compelling: red palm weevil infestation elevates anthocyanin content in palm tissues concurrent with a surge in total phenolics and reactive oxygen species, but mechanical wounding alone can induce part of this accumulation, suggesting a general stress response rather than a specific defensive adaptation (Manzoor et al., 2022). In grapevines, Japanese beetle feeding promotes anthocyanin accumulation, yet transcriptomic data reveal simultaneous upregulation of terpenoid and tannin pathways, making it impossible to attribute resistance specifically to anthocyanins (Calf et al., 2020; Selli et al., 2023). Red galls induced by gall midges on Japanese beech (*Fagus crenata*) exhibit intense pigmentation, but this likely results from localized auxin/cytokinin imbalances rather than a systemic JA/ET defence response, aligning more with a metabolic by-product (Miyazaki et al., 2020).

Critically, the molecular regulatory nodes remain largely opaque in natural systems. No study has measured hormone dynamics (JA/ET titres) in woody ornamentals during natural herbivory, nor has any reported qPCR/transcriptomic data for MBW components specifically induced by chewing insects under field conditions. A recent systematic review on bHLH transcription factors in woody perennials highlights their central role in stress memory, but functional validation under herbivory is absent (Yan et al., 2024b). Furthermore, the functional overlap with proanthocyanidins (PAs) is particularly acute in insect defence: PAs are widely documented as anti-herbivory agents in *Populus* and *Quercus*, and because anthocyanins and PAs share the same upstream pathway (competing for leucoanthocyanidin substrate), observed correlations between red pigmentation and resistance might be indirectly driven by PA flux rather than anthocyanin itself—a possibility that has never been experimentally disentangled in woody ornamentals.

To move beyond correlation, we advocate for hypothesis-driven manipulative experiments: (i) generation of anthocyanin-deficient woody lines (via CRISPR/Cas9 or VIGS targeting *ANS*/*UFGT*) to test H3; (ii) choice-assays with isogenic lines differing only in pigmentation to test H1; and (iii) metabolomic profiling to tease apart anthocyanin-specific versus PA-specific effects. **Table 2** consolidates the current state of knowledge, explicitly marking the substantial gaps in regulation and function.

**Table 2:** Cross-species comparison of insect-induced anthocyanin accumulation across mechanistic nodes in woody perennials, structured by hypothesis support.

Species	Hormone signaling	Transcriptional reprogramming	Structural gene activation	Defensive function & phenotype	Supported hypothesis
Date palm/Red palm weevil	NR (mechanical wounding mimic suggests general stress)	NR	NR	Anthocyanin was induced after infestation; correlation only	H2 (by-product)
Grapevine/ Japanese beetle	NR	NR (transcriptome hints terpenoid/tannin co-induction)	NR	Anthocyanin was induced in infested fruits; correlation only	H2 (by-product/confounded)
Japanese beech/Gall midge	NR (local auxin/cytokinin speculated)	NR	NR	Red gall pigmentation; no functional assay	H2 (local metabolic effect)
Citrus/Asian citrus psyllid	NR (transgenic OE used)	MBW regulators overexpressed (transgenic)	*DFR/RUBY* OE	Reduced host preference & survival; visual deterrence validated	H1 (visual) + H3 (toxicity)
Eucalyptus/Psyllid	NR	NR	NR	Red juvenile leaves correlate with reduced infestation; field behavioral data	H1 (visual)
Horopito/Generalist insects	NR (JA/ET inferred)	NR (anthocyanin content correlated with leaf margin width)	NR	Red leaf margins signal chemical defence; reduced herbivory	H1 (visual)
Conserved core & Key gaps	JA/ET predicted but ZERO hormonal measurements from natural infestations	MBW likely central; ZERO qPCR/RNAseq data from non-transgenic woody systems under natural herbivory	*CHS/DFR* predicted markers; ZERO systemic fold-change data	No loss-of-function studies; no purified anthocyanin feeding assays in any woody ornamental; contribution vs. PAs/terpenoids completely unquantified	H1 (visual) has strongest ecological support; H3 (toxicity) has transgenic evidence but no natural validation; H2 (by-product) plausible but untested

## 4 The anthocyanins synthesis and potential roles under abiotic stresses

### 4.1 Drought stress

Drought triggers osmotic imbalance and a ROS burst in woody plants, yet many ornamental taxa have evolved a conserved adaptive strategy—drought-induced anthocyanin accumulation—that serves dual roles in osmotic adjustment and ROS scavenging (Ma et al., 2025; Xu et al., 2025). Despite this shared phenotypic outcome, the regulatory architecture underlying these processes—perception, signal transduction, and transcriptional activation—remains poorly characterized across species, hampering a coherent understanding of drought adaptation in woody perennials.

A hierarchical signaling cascade can be distilled from the accumulated evidence. Inferred from herbaceous models, under water deficit, osmosensors and turgor changes trigger rapid ABA accumulation (Vo et al., 2026), which, together with ethylene and sometimes JA, forms an upstream hormonal hub. These signals converge on the MBW (MYB-bHLH-WD40) transcriptional complex—the central executioner—which upregulates structural genes (e.g., *F3’H*, *DFR*, *ANS*) to boost anthocyanin production. Downstream, additional layers including non-coding RNAs, histone modifications, and post-translational adjustments fine-tune the amplitude and tissue specificity of the response (Liu et al., 2021). Functionally, this cascade enhances osmotic balance and antioxidant defense, mitigating drought-induced damage.

Empirical support for this cascade reveals a clear pattern: upstream hormonal inputs are species-divergent, whereas the MBW core is universally conserved. Rather than enumerating species-specific cases in isolation, we synthesize the available evidence and reveal that woody ornamentals have evolved at least four distinct upstream strategies to activate the conserved MBW complex under drought. The first strategy is single-pathway reliance, exemplified by crabapple (*Malus spectabilis*), where drought-elicited ethylene exclusively activates *MsERF17*, which directly upregulates the bHLH component and structural genes (Xu et al., 2025). The second is synergistic redundancy, as seen in jujube (*Ziziphus jujuba*), where ABA and ethylene synergistically activate both the *ZjABI5-ZjEIN3* module and a *ZjCaM1-ZjMYB108* cascade, ensuring robust induction under fluctuating stress (Wang et al., 2025a). The third is repressor release, typified by grape, which relies on drought-induced *miR156b* to relieve MBW from repression by *VvSBP* repressors (Guo et al., 2024). The fourth is direct activator induction coupled with downstream sequestration, as observed in rose (*Rosa chinensis*), which directly induces *RcMYB75* as a MBW activator and simultaneously upregulates *RcGSTFL11* to couple anthocyanin synthesis with vacuolar storage (Ma et al., 2025). Thus, while the MBW node acts as a conserved “processing core,” the upstream signals driving it falls into these four mechanistic categories: single-pathway reliance, synergistic redundancy, repressor release, or direct induction—each representing a distinct evolutionary solution to the same selective pressure. This strategy diversification likely reflects adaptive responses to distinct drought regimes encountered during evolutionary history, with species from arid regions favoring synergistic redundancy as a bet-hedging mechanism, whereas domesticated taxa may have streamlined their regulatory networks (Zhang et al., 2025b). A comparative summary of these strategies is provided in **Table 3**.

**Table 3:** Upstream strategies for drought-induced activation of the conserved MBW complex in woody ornamentals.

Species	Primary upstream hormones	MBW activation mode	Key regulators	Parallel regulatory loop	Strategy category
Crabapple (*Malus spectabilis*)	Ethylene	Direct induction of bHLH component	*MsERF17*	None	Single-pathway reliance
Jujube (*Ziziphus jujuba*)	ABA + Ethylene	Synergistic dual-module activation (ABI5-EIN3 + CaM1-MYB108)	*ZjABI5, ZjEIN3, ZjCaM1, ZjMYB108*	None	Synergistic redundancy
Grape (*Vitis vinifera*)	(Inferred: ABA/drought)	Derepression via miRNA-mediated release of MBW from repressors	*miR156b-VvSBP*	None	Repressor release
Rose (*Rosa chinensis*)	(Inferred: drought)	Direct induction of MYB activator coupled with vacuolar sequestration	*RcMYB75, RcGSTFL11*	None	Direct induction + transport coupling
Tree peony (*Paeonia ostii*)	(Inferred: drought)	Indirectly establishing a permissive environment via ROS homeostasis	*PomiR172d-PoARR*	Yes (miRNA-mediated ROS homeostasis)	Parallel-loop assistance

Beyond this core MBW axis, a parallel regulatory loop adds complexity without altering the hierarchical logic. In tree peony (*Paeonia ostii*), drought represses *PomiR172d* to induce its target *PoARR*, which indirectly bolsters ROS homeostasis and creates a permissive cellular environment for anthocyanin accumulation (Li et al., 2026a). This miRNA-mediated module operates in parallel, rather than as a separate hierarchical node. Long non-coding RNAs (lncRNAs) have also been implicated in anthocyanin regulation under stress in woody plants, adding another layer of complexity beyond miRNAs (Xiong et al., 2026). However, major blind spots persist when applying this cascade to woody ornamentals: the initial signal perception node (equivalent to the PYL-SnRK2 receptors in *Arabidopsis*) remains largely uncharacterized in ornamental woody taxa, although recent studies in fruit trees have revealed functional PYL-PP2C-SnRK2 modules regulating anthocyanin accumulation via sugar signaling (Wang et al., 2026). Similarly, the role of epigenetic chromatin remodeling in stress memory and transcriptional priming remains relatively unexplored (Siddique et al., 2024). Moreover, the temporal dynamics—whether early versus late drought, or mild versus severe stress, engages the same nodes—also remain unexplored.

Critically, the field must transcend the current reliance on heterologous overexpression in herbaceous models and phenotypic correlations. Future validation requires: (i) direct loss-/gain-of-function assays in woody tissues via VIGS or agroinfiltration to establish causality—approaches validated in species such as blueberry, tree peony, and red-skinned pear for anthocyanin-related genes (Han et al., 2022; Tian et al., 2023; Yang et al., 2024a); (ii) time-resolved multi-omics (transcriptome-metabolome-chromatin) to resolve the order of events and pinpoint early vs. late regulators; and (iii) comparative quantitative modeling across contrasting drought-tolerant species to test whether the cascade is universally applicable or subject to ecological specialization. Addressing these priorities will transform our view of anthocyanin-mediated drought adaptation from a collection of species-specific anecdotes into a predictive, systems-level framework.

### 4.2 Salt stress

Salt stress imposes ionic toxicity and osmotic imbalance (Zhu, 2002), eliciting Ca²^+^ spikes and ROS bursts that rapidly promote ABA and ethylene accumulation, forming an upstream hormonal hub. This hub converges on the conserved MYB-bHLH-WD40 (MBW) transcriptional complex, which subsequently upregulates structural genes (*CHS*, *DFR*, *ANS*) and vacuolar transporters (e.g., *GST*) to enhance anthocyanin production and vacuolar sequestration. Functionally, anthocyanins act as ROS scavengers and ion chelators, mitigating salt-induced oxidative damage and Na^+^ toxicity (Mo et al., 2022; Sun et al., 2022; Hu et al., 2024). This core cascade is analogous to that observed in the drought response, but salt-specific features—particularly the prominent roles of Ca²^+^ signaling and direct ion-chelation—distinguish the regulatory logic.

Despite a shared MBW core, woody ornamentals have evolved diverse upstream strategies to drive anthocyanin biosynthesis under salt stress. These strategies can be categorized into four mechanistic classes, which are not mutually exclusive but rather reflect the varying regulatory priorities across species.

(i) Metabolic flux reprogramming – In grape (*Vitis vinifera*) roots, short- and long-term salt stress differentially shift carbon allocation through the flavonoid pathway, with coordinated up-regulation of *DFR*, *ANS*, and *FLS* leading to accumulations of leucoanthocyanidins and anthocyanins (Chen et al., 2025c). This strategy does not necessarily alter the core MBW regulators; instead, it adjusts precursor availability and pathway branching, thereby rerouting metabolic flux into the anthocyanin branch. This provides the quantitative basis for subsequent production. By re-routing carbon skeletons from primary metabolism into secondary metabolism, this route lays the quantitative groundwork for subsequent anthocyanin production.

In parallel with this metabolic foundation, hormonal signals modulate the response amplitude of the flux-driven response. (ii) Hormonal synergy and exogenous signal amplification – In apple calli, *MdGA2ox7* cooperates with melatonin to boost anthocyanin biosynthesis and alleviate salt/cold injury (Yan et al., 2024a). This strategy operates as a signal-amplifying module, likely through crosstalk with the ABA/ethylene hormonal hub, adjusting the sensitivity and magnitude of MBW activation without introducing a qualitatively new regulatory direction. Together with metabolic reprogramming, this auxiliary layer ensures that the biosynthetic pathway is not only supplied with precursors but also dynamically tuned to stress intensity.

Although metabolic flux and hormonal amplification set the stage, the most direct and conserved route entails reinforcing the MBW transcriptional core itself. (iii) Transcriptional boost of MBW output – This route operates by directly increasing the abundance or activity of MBW components. In apple (*Malus domestica*), the calcium–ethylene signaling module exemplifies this mechanism: *MdVQ8* promotes *MdWRKY75*-mediated activation of *MdMYB1* and stabilizes the *MdTCP46–MdMYB1* interaction, while *MdCML15* further stabilizes these transcriptional complexes. Conversely, *MdEBF*1 antagonizes this route by facilitating *MdVQ8* degradation, thus forming a dynamic *MdEBF1-MdVQ8-MdCML15* regulatory loop that fine-tunes MBW output under salt stress (Gao et al., 2026). A conceptually similar scenario is observed in areca palm (*Areca catechu*), where salt-inducible *AcMYB176* directly upregulates *AcCHS5*, enhancing both anthocyanin production and antioxidant enzyme activities (Jiang et al., 2025). Collectively, these examples illustrate that direct potentiation of the MBW transcriptional machinery represents the most widely adopted strategy across woody ornamentals.

Once synthesized via the aforementioned pathways, the protective function of anthocyanins ultimately depends on chemical stability and subcellular compartmentation—a downstream layer of MBW activity. (iv) Post-modification and vacuolar sequestration – In *Rhododendron agastum*, the UDP-glycosyltransferase (UGT) *Ra3GT2* (a flavonoid 3-O-glycosyltransferase) modifies anthocyanins and flavonols, enhancing their stability and aqueous solubility. Functional evidence confirms that *Ra3GT2* overexpression confers salt/drought tolerance, whereas the *ugt78d2* mutant displays increased sensitivity (Luo et al., 2025). This strategy thus operates at the post-synthetic level, ensuring that once anthocyanins are produced, they are efficiently stabilized and stored to perform their ROS-scavenging and ion-chelating roles. As the final step in this biosynthesis-to-sequestration cascade, post-modification and sequestration dictate the overall physiological output of the pathway.

Beyond these direct activatory routes, epigenetic modifications—including DNA methylation, histone marks (H3K9ac, H3K4me3, H3K27me3), and non-coding RNAs—have been implicated in salt-induced gene regulation in herbaceous model plants and annual crops (Zhang et al., 2024a). However, direct evidence in woody ornamentals remains scarce; bivalent H3K4me3-H3K27me3 marks and methyltransferase/demethylase dynamics (Nunez-Vazquez et al., 2022) offer promising avenues but await functional validation in perennial systems. These epigenetic layers likely act as modulators of response amplitude and memory rather than as primary on-off switches.

**Table 4** summarizes the contrasting strategies and key regulatory components across species, highlighting that while the MBW complex serves as the universal executioner, the upstream input—whether via transcriptional activation, post-synthetic modification, flux reallocation, or hormonal synergy—varies substantially. Notably, calcium signaling appears particularly prominent in apple, whereas post-modification dominates in Rhododendron, and metabolic reprogramming is evident in grape. Future research should prioritize: (i) direct loss-/gain-of-function assays in woody tissues to establish causality, (ii) time-resolved multi-omics to dissect early vs. late salt responses, and (iii) comparative modeling across salt-tolerant and sensitive species to test whether the same strategies predict ecological adaptation.

**Table 4:** Comparative overview of salt-induced anthocyanin regulatory strategies in woody ornamental plants.

Representative species	Key regulator(s)	Functional layer	MBW relationship	Strategy type
Apple (*Malus*)	*MdVQ8-MdWRKY75-MdMYB1, MdTCP46, MdCML15, MdEBF1*	MBW activator complex stabilization	Direct upregulation	Transcriptional enhancement
Areca palm (*Areca catechu*)	*AcMYB176 → AcCHS5*	Transcriptional activation of structural gene	Direct upregulation	Transcriptional enhancement
*Rhododendron agastum*	*Ra3GT2* (glycosyltransferase)	Anthocyanin modification and stability	Downstream of MBW	Post-modification & sequestration
Grape (*Vitis*)	*DFR, ANS, FLS* (differential expression)	Carbon flux allocation within flavonoid pathway	Upstream of MBW (flux rerouting)	Metabolic flux reprogramming
Apple calli	*MdGA2ox7* + melatonin	Signal amplification and stress alleviation	Auxiliary to MBW	Hormonal synergy

### 4.3 Low temperature stress

Low temperature triggers reactive oxygen species (ROS) accumulation and membrane lipid peroxidation in woody ornamental plants, compromising their visual quality (Yang et al., 2016). In response, many species enhance anthocyanin biosynthesis, which serves as an antioxidant and membrane stabilizer to alleviate cold-induced damage (Zhang et al., 2023; Chen et al., 2024a). Although cold effectively induces anthocyanin production across diverse taxa (Yu et al., 2022), the regulatory framework underlying this conserved response can be distilled into several functional modules that operate in parallel or in series, rather than as a collection of species-specific idiosyncrasies. A general working model emerges from the cumulative evidence: cold signals are perceived (partly via the ICE-CBF cascade, a conserved cold-sensing module well-characterized in herbaceous models but increasingly evident in woody taxa) (Sang et al., 2025) and transduced through three core regulatory layers—(i) transcriptional reprogramming via direct activation of MYB/bHLH/NAC transcription factors and the MBW complex; (ii) epigenetic and metabolic remodeling, including promoter demethylation, sugar signaling, and vacuolar sequestration; and (iii) hormone signal crosstalk, particularly with ABA, brassinosteroids, and ethylene. Superimposed on these positive regulators, a negative feedback layer involving ubiquitin-mediated degradation and SnRK-MYBS3 modules fine-tunes the amplitude to avoid overaccumulation. Below, we dissect how different woody ornamentals engage these modules, highlighting mechanistic convergence despite divergent upstream effectors.

Transcriptional activation of the core biosynthetic machinery constitutes the primary driver. In Malus ‘Pinkspire’ fruit, cold-induced *MpYABBY2* promotes ABA synthesis and upregulates *MpMYB11*, which together with *MpbHLH79* forms an MBW complex to activate downstream structural genes (Chen et al., 2025b). Similarly, in *Acer pseudosieboldianum*, *ApseMYB16* and *ApseMYB31* are strongly upregulated (52-fold and 28-fold) under cold, correlating with leaf reddening (Zhang et al., 2025a). In pear, *PyNAC42* interacts with *PyMYB10* to synergistically enhance the transcription of anthocyanin genes (Zhang et al., 2024b). In contrast, paper mulberry deploys a more direct route: cold directly induces BpCHS2, and its overexpression suffices to boost anthocyanin accumulation and cold tolerance (Fu et al., 2025). Thus, while the MBW/NAC node is universally targeted, the upstream activators vary from YABBY-MYB cascades to direct CHS induction.

Epigenetic and metabolic modifications provide a second layer of regulation that modulates the accessibility and efficiency of the biosynthetic pathway. In apple, low temperature reduces the methylation levels at the *MdCHS* and *MdUFGT* promoters, relieving epigenetic repression and allowing transcriptional activation (Yu et al., 2022). In paper mulberry, cold induces *BpETR2* to promote sugar synthesis, while *BpGST* enhances anthocyanin transport to vacuoles, coupling biosynthesis with storage (Fu et al., 2025). These modifications ensure not only increased production but also proper compartmentalization and stability of anthocyanins. Importantly, histone modifications (e.g., H3K9ac, H3K4me3, H3K27me3) and non-coding RNAs also contribute to this regulatory layer by modulating chromatin state and post-transcriptional turnover, though their roles in woody cold responses are less well characterized than in herbaceous systems (Yu et al., 2022; Wang et al., 2025c).

Hormonal crosstalk further integrates cold signals with endogenous development cues. In blood orange (an evergreen tree grown both for ornament and fruit) exposure to 8 °C activates brassinosteroid and ethylene signaling, which in turn upregulates a suite of structural genes including *PAL*, *CHS*, *DFR*, and *UFGT* (Chen et al., 2025a). This suggests that hormones act as intermediate relays, amplifying the cold signal and coordinating it with other stress responses.

Negative regulatory mechanisms are equally important to prevent excessive anthocyanin accumulation under prolonged cold. In red maple, cold-induced ArMYB89 directly activates the glycosyltransferase gene *ArUGT52*, but the E3 ubiquitin ligase *ArCOP1* mediates *ArMYB89* degradation, thereby attenuating the response (Shah et al., 2024). In paper mulberry, *BpSnRK* and *BpMYBS3* act as negative regulators, adding another layer of fine control (Fu et al., 2025). This balance between activation and repression ensures an optimal anthocyanin level for oxidative protection without minimizing metabolic cost. To provide a clear comparative overview, we summarize the key regulatory modules and their representative species in **Table 5**.

**Table 5:** Functional modules and representative regulators involved in low-temperature-induced anthocyanin accumulation in woody ornamental plants.

Representative species	Key regulators	MBW relationship	Functional role	Regulatory module
*Malus* ‘Pinkspire’, *Acer pseudosieboldianum*, Pear	*MpYABBY2-MpMYB11-MpbHLH79; ApseMYB16/31; PyNAC42-PyMYB10*	MBW-dependent (core complex directly drives structural genes)	Establishes the primary biosynthetic flux by directly upregulating rate-limiting enzymes (*CHS*, *DFR*, *UFGT*)	Transcriptional activation (MBW/NAC/YABBY)
Paper mulberry	*BpCHS2*	MBW-independent (bypasses canonical MBW; upstream positive MYB not characterized, while BpMYBS3 acts as negative regulator)	Bypasses MBW, directly upregulates a key enzyme in the flavonoid pathway	Direct structural gene induction
Apple	*MdCHS*/*MdUFGT* promoter demethylation; histone marks (H3K9ac/H3K27me3)	Modulates MBW target accessibility (removes silencing to permit MBW binding)	Removes chromatin silencing; modulates chromatin accessibility and transcript stability	Epigenetic de-repression & chromatin remodeling
Paper mulberry	*BpETR2* (sugar synthesis), *BpGST* (vacuolar transport)	Parallel to MBW (acts independently to stabilize and store end products)	Enhances precursor supply and sequesters anthocyanins for stability	Metabolic coupling (sugar signaling & transport)
Blood orange	BR and ethylene signaling pathways	Upstream of MBW (amplifies signal and upregulates MBW targets)	Amplify cold signal and upregulate multiple structural genes (PAL, CHS, DFR, UFGT)	Hormonal relay (BR/ethylene)
Red maple, Paper mulberry	*ArCOP1-ArMYB89*; *BpSnRK-BpMYBS3*	Suppresses MBW output (direct degradation of MBW activators or downstream effectors)	Fine-tune response duration and amplitude, prevent overaccumulation	Negative feedback (ubiquitination/SnRK)

Collectively, this cross-species comparison reveals that cold-induced anthocyanin synthesis in woody ornamentals is not governed by a single, species-restricted pathway, but rather by a set of conserved functional modules—transcriptional activation, epigenetic remodeling (including histone marks and non-coding RNAs), hormonal integration, and negative feedback. Different species recruit different upstream factors (e.g., MYB, NAC, YABBY, or direct CHS induction) to engage these modules, reflecting evolutionary flexibility at the input level while maintaining a common output logic. Future work should focus on how these modules are temporally coordinated and how their crosstalk is fine-tuned under varying cold intensities and durations, using multi-omics and CRISPR-based editing in woody tissues.

### 4.4 High temperature stress

High temperature is one of the major abiotic stresses limiting the growth and ornamental quality of woody ornamental plants. In contrast to low-temperature-induced anthocyanin accumulation, high temperature generally suppresses anthocyanin biosynthesis in woody ornamental plants, leading to poor coloration and reduced quality (Meng et al., 2015; Movahed et al., 2016). A general hierarchical framework can be distilled from recent advances: high-temperature signals are perceived through multiple sensors, including phytochrome-mediated thermosensing and ER-resident unfolded protein response (UPR) sensors, such as *VvbZIP17* in grape (Sun et al., 2025b), which trigger hormone reprogramming (particularly auxin and brassinosteroid signaling) and protein homeostasis perturbations. These upstream events converge on three core execution modules—(i) transcriptional reprogramming via the induction of multiple transcriptional repressors; (ii) post-translational modulation of regulatory protein stability; and (iii) ER-stress-mediated metabolic flow diversion—which converge to suppress the MBW complex activity or direct downregulation of structural anthocyanin genes (e.g., *ANS*, *UFGT*). Parallel to this suppression axis, high temperature also upregulates degradation-related enzymes (e.g., polyphenol oxidases, peroxidases) that dismantle accumulated anthocyanins, adding a post-synthetic loss pathway that operates independently of the MBW regulatory logic. Superimposed on these modules, tissue-specific regulatory circuits add an additional layer of spatial precision. Below, we dissect how different woody ornamentals engage these modules, revealing that while upstream effectors are species-divergent, the functional logic of targeting the anthocyanin production and stability node is evolutionarily conserved.

Transcriptional repressor networks constitute the first core module. In apple, high temperature upregulates *MdBZR1* and *MdLBD37*, which form a positive feedback loop that synergistically inhibit anthocyanin synthesis (Li et al., 2025a). Concurrently, *MdGATA15* is activated under high temperature; it directly represses the activator *MdMYB11*, the structural gene *MdANS*, and the transporter gene *MdGSTF12*, while activating the repressor *MdMYB308*, thereby establishing a multi-layered transcriptional inhibitory network (Fang et al., 2025). In grape, similar repressive logic is executed by *VvMYB44-1*, which directly suppresses the MBW activator *VvMYBA1* and structural genes under high-temperature conditions, while *VvLBD11* and *VvLBD28* add further layers of inhibition on both biosynthesis and vacuolar transport (Feng et al., 2025; Leng et al., 2025). This module operates by directly suppressing both the MBW complex components and their downstream targets at the transcriptional level.

Post-translational regulation—specifically modulation of repressor protein stability—provides a second, distinct module. In pear, high temperature reduces the ubiquitination level of *PpHsfB2a*, leading to its protein accumulation. This accumulated *PpHsfB2a* subsequently shuts off light signaling—through interference with *PpHY5L*—to inhibit anthocyanin synthesis (Wang et al., 2025d). Unlike the transcriptional module, this mechanism rapidly fine-tunes the response through ubiquitin-proteasome system (UPS)-mediated control without altering transcript levels of the core machinery.

Hormone-ER stress crosstalk represents a third mechanistic module that operates upstream of structural genes. In grape, high-temperature-induced elevation of auxin levels triggers ER stress via the *VvARF3-VvFHY3* interaction and directly downregulate anthocyanin biosynthetic genes (Sun et al., 2025b). Notably, a positive regulatory pathway also exists in grape—*VvBBX32* activates *VvBZR1* to promote anthocyanin accumulation under specific high-temperature regimes (Qiu et al., 2025). This coexistence of opposing outputs within the same species suggests that the grape anthocyanin network may integrate both intensity-dependent and stage-dependent gating mechanisms, rather than functioning as a unidirectional suppressive switch. The conditions that determine whether the positive or negative arm prevails remain unresolved.

In addition to the three core suppressive modules, high temperature promotes anthocyanin degradation. In *Loropetalum chinense* var. *rubrum*, elevated temperature upregulates *LcWRKY18*, which directly activates the degradation-related gene *LcPPO*, accelerating pigment breakdown and contributing to the “regreening” phenotype (Huang et al., 2026). This degradation module operates in parallel to the synthetic suppression modules, jointly determining the net pigmentation outcome under heat stress. Tissue-specific regulatory differentiation adds a fourth layer of spatial complexity without altering the core inhibitory logic. In peach (*Prunus persica*), *PpBBX32* regulates anthocyanin accumulation in the peel, whereas *PpZAT5* does so in the flesh (Huang et al., 2024). This indicates that even within the same species and under identical temperature conditions, different organs recruit distinct regulators to fine-tune suppression patterns, likely reflecting differential resource allocation strategies.

Collectively, to provide a clear comparative overview, we summarize the key regulatory modules, their representative species, and their mechanistic logic in **Table 6**. This cross-species comparison reveals that high-temperature suppression of anthocyanin biosynthesis in woody ornamentals is not a set of unrelated phenomena. Instead, it operates through a limited set of conserved functional modules that target the same ultimate node—the MBW complex and/or its downstream structural targets—yet differ in their mechanistic entry points. Specifically, species have evolved three convergent strategies: (i) transcriptional annihilation of MBW components and structural genes; (ii) post-translational stabilization of negative regulators that intercept upstream signals; and (iii) hormone-driven ER stress that redirects cellular metabolism away from anthocyanin production. A fourth, mechanistically distinct layer—active degradation—operates post-synthetically, ensuring that net pigmentation reflects the balance between synthesis and breakdown. Superimposed on these, tissue-specific regulators ensure that the suppressive logic is deployed with spatial precision. Notably, this modular architecture stands in stark contrast to the induction mechanisms observed under drought and low temperature, where MBW activation rather than suppression is the dominant output. Major unresolved questions include how the conditional positive pathway in grape is gated, whether the ER-stress and degradation modules are universally deployed across woody taxa, and how these modules are temporally coordinated under fluctuating temperature regimes. Addressing these priorities will require time-resolved multi-omics and direct causal tests via VIGS or CRISPR-based editing in woody tissues, moving the field from descriptive correlations to predictive mechanistic frameworks.

**Table 6:** Functional modules and representative regulators involved in high-temperature-induced suppression of anthocyanin accumulation in woody ornamental plants.

Representative species	Key regulators	Impact on MBW	Functional role	Regulatory module
Apple (*Malus* spp.), Grape (*V. vinifera*)	*MdBZR1-MdLBD37*; *MdGATA15→MdMYB308*; *VvMYB44-1*; *VvLBD11/28*	Directly suppresses MBW activators and structural genes while boosting repressors	Establishes multi-layer transcriptional barriers to prevent MBW assembly	Transcriptional repressor networks
Pear (*Pyrus* spp.)	*PpHsfB2a* (reduced ubiquitination → accumulation)	Indirectly suppresses MBW by shutting off upstream light signaling (*PHY5L*)	Enables rapid, transcription-independent fine-tuning via UPS	Post-translational stabilisation of repressors
Grape (*V. vinifera*)	Auxin → *VvARF3*-*VvFHY3* → ER stress (suppression); *VvBBX32*-*VvBZR1* (conditional activation)	Directly downregulates structural genes (e.g., *ANS*, *UFGT*); positive arm may act independently	Conditionally deploys opposing outputs depending on stress intensity/stage	Hormone-ER stress crosstalk
Loropetalum (*Loropetalum chinense* var. *rubrum*)	*LcWRKY18*→*LcPPO*	MBW-independent (operates post-synthetically)	Accelerates enzymatic breakdown of existing anthocyanins	Degradation promotion
Peach (*Prunus persica*)	*PpBBX32* (peel) vs. *PpZAT5* (flesh)	Differentially regulates the same pathway in distinct organs	Ensures spatial adaptation and optimizes organ-specific resource partitioning	Tissue-specific differentiation

### 4.5 Heavy metal stress

Heavy metal ions trigger phytotoxic effects in woody ornamentals—chlorophyll degradation, membrane lipid peroxidation, ion imbalance, and ROS burst (Gomes et al., 2017; Singh et al., 2021). Many woody taxa employ anthocyanins as a first line of defense (Imtiaz et al., 2018; Li et al., 2021b; Altaf et al., 2024). Unlike drought or cold, where anthocyanins act primarily as antioxidants, under heavy metal stress they acquire unique dual functionality. Their 3′,4′-dihydroxy B-ring chelates metal ions to form stable complexes, reducing free cation bioavailability (Cherrak et al., 2016; Fedenko et al., 2017; Maleva et al., 2018; Estévez et al., 2021). These complexes are then sequestered into vacuoles, removing them from the cytosol and other metabolic sites (Ahammed and Yang, 2022). This two-step strategy—chelation plus compartmentalization—defines heavy metal detoxification in perennials. Cumulative evidence from diverse woody ornamental plants allows the synthesis of a general regulatory framework comprising four functional modules that operate either in parallel or in sequence. These modules are: (i) signal perception and early ionic/redox events, which couple metal entry to downstream transcriptional reprogramming; (ii) transcriptional activation of the anthocyanin biosynthetic pathway, primarily mediated by the MBW (MYB-bHLH-WD40) complex; (iii) transporter-dependent vacuolar sequestration, which removes metal–anthocyanin complexes from the cytosol; and (iv) a regulatory layer that is both dose-dependent and modulated by exogenous signals, and fine-tunes the intensity of the response. Despite species-specific effectors, the module logic is conserved.

Signal perception is the least characterized module. Unlike drought (PYL receptors) or cold (ICE1-CBF), metal sensors in woody taxa remain largely unknown. Ca²^+^ influx, ROS waves, and MAPK cascades likely serve as early transducers (Li et al., 2026c). In *Liriodendron* hybrid, zinc-induced *LhCAT2* links ROS homeostasis to anthocyanin biosynthesis (Ke et al., 2026). In apple, the *miR408a*-*BBP*-*LAC3*/*CSD1* module perceives copper and integrates it with light signaling (Hu et al., 2023). These examples suggest a redox-MAPK-miRNA relay converging on MBW activation.

Transcriptional activation is the second committed step, with negative feedback serving as its intrinsic brake. In poplar, Cd upregulates *CHS*, *F3′H*, *DFR*, and *ANS*, enhancing metal tolerance (Sun et al., 2025a). In *Lycium ruthenicum*, the R2R3-MYB activator *LrAN2* drives both anthocyanin biosynthesis and the detoxification cascade (Ai et al., 2025). MBW is thus the central hub, though upstream activators involved in the metal stress response are less well characterized than those in drought or cold (Hu et al., 2023). To prevent overaccumulation, negative feedback operates in parallel: in red maple, *ArCOP1* ubiquitinates the MYB activator *ArMYB89* (Shah et al., 2024); MYB repressors and ubiquitin-mediated turnover likely acts as general negative regulators of MBW output (An et al., 2017), though metal-specific repressors remain to be identified.

Vacuolar sequestration via transporters constitutes a functionally distinct module. Plants actively transport metal–anthocyanin complexes into vacuoles (Ahammed and Yang, 2022). In *L. ruthenicum*, *LrAN2* directly activates the MATE transporter *LrTT12*; this axis is essential for Cd tolerance (Ai et al., 2025). MATE/ABC transporters may serve as general carriers—blueberry *VcMATE1/4* are implicated in xenobiotic detoxification and cation export (Chen et al., 2015)—though substrate specificities need further study.

Dose-dependent modulation and exogenous signals together tune response intensity. In the poplar cultivar ‘Quanhong’, mild Cd (50 μM) promotes growth and anthocyanin enzyme activity, while high Cd (100–150 μM) suppresses both (Zhang et al., 2014); similar trends occur in *Berberis thunbergii* (Chadzinikolau et al., 2017). This “low-promotion, high-inhibition” pattern reflects metabolic trade-offs, but the molecular sensors remain unknown. Exogenous signals can potentiate this system: melatonin upregulates *C4H*, *LAR*, and *ANS* in tea plants, enhancing Pb tolerance in high-anthocyanin genotypes (Li et al., 2025b); NO and brassinosteroids also amplify anthocyanin accumulation under metal stress (Chen et al., 2015).

In summary, **Table 7** summarizes the key features. Heavy metal detoxification in woody ornamentals is governed by four conserved modules—perception, MBW activation/feedback, vacuolar sequestration, and intensity tuning—rather than species-specific idiosyncrasies. Unlike drought/cold, anthocyanins here serve in chelation and compartmentalization in addition to antioxidation. Metal-specific MYB activators, transporter coupling, and dose-sensitive sensors likely underpin this regulatory distinction (Zheng et al., 2022). This positions the anthocyanin pathway as a promising phytoremediation target. Future work should identify upstream metal sensors, characterize transporter specificity, and develop strategies that coordinately enhance biosynthesis and sequestration.

**Table 7:** Functional modules involved in heavy-metal-induced anthocyanin-mediated detoxification in woody ornamental plants.

Representative species	Regulatory module	Key regulators	Impact on MBW	Functional role
Liriodendron hybrid; Apple (*Malus*)	Signal perception & early signaling	*LhCAT2*; *miR408a*-*BBP-LAC3/CSD1*	Upstream of MBW (redox/MAPK/miRNA relay converges on MBW activation)	Couples metal-triggered ROS/Ca²^+^ changes to transcriptional reprogramming
Poplar *(Populus*); *Lycium ruthenicum*; Red maple (*Acer rubrum*)	Transcriptional activation + Negative feedback	*LrAN2* (MYB activator); *ArCOP1* (E3 ubiquitin ligase)	Directly drives MBW output; ubiquitination suppresses MBW activator stability	Activates structural genes (CHS, F3′H, DFR, ANS) for anthocyanin biosynthesis; prevents overaccumulation via degradation of MYB activators
Lycium ruthenicum; Blueberry (*Vaccinium*)	Vacuolar sequestration	*LrAN2→LrTT12* (MATE transporter); *VcMATE1/4*	Downstream of MBW (transporters are targets of MBW or functionally parallel)	Physically removes metal–anthocyanin complexes from cytosol into vacuoles
Poplar ‘Quanhong’; *Berberis thunbergii*; Tea (*Camellia sinensis*)	Dose-dependent modulation + Exogenous signal tuning	Melatonin-induced *C4H*/*LAR*/*ANS*; NO/BR signaling	Modulates MBW output intensity (biphasic response; pharmacological priming)	Adjusts detoxification investment to stress severity; amplifies overall efficiency via exogenous signals

### 4.6 Light stress

Light is an indispensable energy source for photosynthesis, but excess radiation (e.g., high light intensity, UV-B) can cause photoinhibition and photo-oxidative damage (Croce and Amerongen, 2014). Under high light and UV-B stress, anthocyanins protect photosynthetic pigments by absorbing excess light energy (especially in the green spectrum of 500–600 nm) and UV radiation, and they directly scavenge reactive oxygen species, thereby establishing a “shading-antioxidant” dual defense mechanism (Takahashi and Badger, 2011).

Despite the diversity of light signals (intensity, quality, and UV-B) and the variety of woody ornamental species studied, a general regulatory framework can be distilled from accumulating evidence. Light signals are perceived by specific photoreceptors—UVR8 for UV-B, phytochromes (Phy) for red/far-red, and cryptochromes (Cry) for blue light—which trigger a hierarchical signaling cascade. This cascade converges on four interconnected functional modules: (i) energy-sensing and phosphorylation signaling (e.g., SnRK1 kinases), which couples cellular energy status to transcriptional reprogramming; (ii) photoreceptor-specific transcriptional modules, where different light qualities activate distinct sets of transcription factors (BBXs, MYBs, WRKYs) that either directly induce or derepress the MBW complex; (iii) hormone-crosstalk and downstream modification modules, which integrate light with ABA, gibberellin, or other hormonal signals and ensure proper glycosylation and vacuolar sequestration of anthocyanins; and (iv) negative feedback regulation, which acts through activator-repressor antagonism (e.g., *HY5* vs. *PIFs*, *MYB12* vs. *MYB4*) and ubiquitination-mediated degradation (e.g., *COP1*-*HY5*) to fine-tune response amplitude and prevent overaccumulation. We analyze how different woody ornamentals engage these modules, highlighting mechanistic convergence despite divergent upstream effectors.

Energy-sensing and phosphorylation signaling module. Under high-light acclimation, cellular energy status is monitored by SnRK1 kinases, which directly phosphorylate and activate the *PAP1* transcription factor (a key MBW component). This mechanism has been functionally validated in poplar (*Populus* spp.), where *SnRK1*-mediated PAP1 activation enhances both anthocyanin accumulation and photoprotection (Dieckmann et al., 2025).

Photoreceptor-specific transcriptional modules. Different light qualities engage distinct photoreceptors yet all converge on the MBW complex. Blue light induces *MiBBX24/27*-*MiMYB1* in mango (Pan et al., 2025), while in poplar and pear, *PtrBBX23*-HY5 and *PpBBX18* function as indispensable regulators for light-induced anthocyanin biosynthesis (Li et al., 2021a; Zhang et al., 2022b). In grape, red and blue light activate ABA signaling via *VvPHY* and *VvCRY*, upregulating *VvSnRK2.6*/*VvABF2* and promoting *VvMYBa1*-*VvUFGT* expression (Kondo et al., 2025). UV-B, perceived by UVR8, operates through multiple routes: the *CpMYB12*/*CpMYB4* antagonistic pair in wintersweet (Wang et al., 2025f); the *PcWRKY11*-*PcPIF3-PcMYB10* cascade in pear (Cao et al., 2026); and *MdWRKY71-L*/*72*-*MdHY5*-*MdMYB1* in apple, and downstream glycosylation via *UGT79B1* in blood orange (Su et al., 2022).

Hormone-crosstalk and downstream modification module. Light signals are integrated with endogenous hormones and post-translational modifications. In sweet cherry, the *PavDREB1E*-*PavD8*-*PavHY5* module integrates light and gibberellin signals (Xiao et al., 2025). Glycosylation and vacuolar transport are essential for anthocyanin stability; UV-B-induced *UGT79B1* enhances glycosylation, increasing pigment solubility and antioxidant capacity (Yang et al., 2025).

Negative feedback as a pervasive fine-tuning layer. The COP1-HY5 module serves as a conserved negative regulatory axis: COP1 degrades HY5 in darkness, while light suppresses COP1 activity to stabilize HY5 and promote anthocyanin accumulation. The antagonistic logic extends to MYB activators and repressors—exemplified by *CpMYB12*/*CpMYB4* in wintersweet (Wang et al., 2025f)—and *PIF*-mediated repression opposing *HY5* activation. Time-resolved transcriptomics in pear peels has revealed a biphasic response, with genes at 6 h being associated with photoreceptor signaling, whereas those at 144 h coincide with peak anthocyanin accumulation, highlighting temporal complexity that remains largely unexplored in woody taxa (Bai et al., 2017).

Collectively, we summarize the key regulators and their relationships to the MBW complex in **Table 8**. This cross-species comparison reveals that light-induced anthocyanin synthesis in woody ornamentals is governed by a set of conserved functional modules—energy-sensing phosphorylation, photoreceptor-specific transcriptional activation, downstream glycosylation/hormonal integration, and negative feedback—with the COP1-HY5-PIFs hub serving as a central regulator. Different species recruit distinct upstream factors to engage these modules, reflecting evolutionary flexibility at the input level while maintaining a common photoprotective output logic. Future work should focus on temporal coordination across varying light conditions, particularly the transition from early signaling to late biosynthetic phases, using time-resolved multi-omics and CRISPR-based validation in woody tissues.

**Table 8:** Functional modules and representative regulators involved in light-induced anthocyanin accumulation in woody ornamental plants.

Representative species	Regulatory module	Signal/photoreceptor	Key regulators/targets	Relationship to MBW complex	Functional outcome
Poplar	Energy‐sensing & phosphorylation	High light	SnRK1 → *PAP1*	Directly activates MBW activator	Rapid initiation of anthocyanin synthesis
Mango	Blue‐light‐specific activation	Blue light/Cry	*MiBBX24/27* → *MiMYB1*	Upstream of MBW	Activates anthocyanin and carotenoid pathways
Poplar, Pear	Blue/high‐light via BBX‐HY5	Blue/high light	*PtrBBX23*‐*HY5*; *PpBBX18*	Interacts with *HY5* to enhance MBW activation	Light‐induced anthocyanin accumulation
Grape	Red/blue light via ABA relay	Red/blue light/Phy & Cry	*VvSnRK2.6*/*VvABF2* → *VvMYBa1*	ABA‐mediated MBW activation	Synergistic anthocyanin accumulation
Wintersweet	UV‐B via activator‐repressor balance	UV‐B/UVR8	*CpMYB12* vs. *CpMYB4*	Modulates MBW via antagonistic TFs	Fine‐tunes anthocyanin under UV‐B
Pear	UV‐B via WRKY‐PIF‐MYB	UV‐B/(UVR8)	*PcWRKY11* → *PcPIF3* → *PcMYB10*	Directly activates MBW activator	Promotes anthocyanin biosynthesis
Apple	UV‐B via WRKY‐HY5‐MYB	UV‐B/UVR8	*MdWRKY71‐L/72* → *MdHY5* → *MdMYB1*	Activates MBW via HY5‐MYB cascade	UV‐B‐induced anthocyanin accumulation
Blood orange	UV‐B via glycosylation	UV‐B/UVR8	*UGT79B1*	Downstream of MBW	Increases anthocyanin stability
Sweet cherry	Light‐hormone integration	Light + GA	*PavDREB1E*‐*D8‐*HY5	Upstream of MBW	Coordinated regulation
Poplar, Pear	Negative feedback	Light/COP1‐HY5	COP1 → HY5 *degradation*	Suppresses MBW output via HY5 destabilization	Prevents overaccumulation

### 4.7 Other stresses

#### 4.7.1 Mechanical damage

Mechanical damage (e.g., pruning, abrasion, and vibration during transport) activates multilayered defense responses in woody ornamental plants. From the accumulated evidence, a general working model can be distilled: mechanical signals are primarily transduced through two distinct mechanistic modules that converge on the MBW core—(i) the jasmonate (JA)-dependent module, where wounding triggers JA biosynthesis, leading to the degradation of JAZ repressors and the subsequent release of the MBW complexes, and (ii) the direct transcriptional activation module, where wound-induced ROS/calcium signals directly activate specific transcription factors (e.g., WRKYs) that either upregulate structural genes or synergistically enhance JA signaling. These modules subsequently drive anthocyanin accumulation, whereas excessive or sustained mechanical force (e.g., strong vibration) can physically disrupt preformed anthocyanin polymers, causing color fading—a post-synthetic effect distinct from transcriptional regulation.

These mechanistic modules are well illustrated by species-specific cases. In poplar, wounding induces JA accumulation, which promotes the degradation of *PtrJAZ1* and thereby releases the *PdMYB118*-*PdTT8* complex to activate downstream anthocyanin biosynthetic genes (Wang et al., 2020)—a canonical example of the JA-dependent module. In grape, wound-induced *VvWRKY5* operates at the intersection of both modules: it directly binds to the *VvUFGT* promoter to activate transcription, while concurrently binding to the *VvLOX* promoter to boost JA production, creating a positive feedback loop that synergistically amplifies anthocyanin accumulation (Zhang et al., 2024c). These cases demonstrate that while the MBW complex remains the conserved execution hub, different woody species have evolved either a singular reliance on JA derepression or a combined WRKY-JA feed-forward strategy to tailor the response to mechanical cues. Importantly, strong or sustained vibration (e.g., during transport) has been reported to dissociate anthocyanin polymers, leading to color fading independently of transcriptional changes (Pérez-Pérez et al., 2018; Flores-Sosa et al., 2021), indicating that the net phenotype is determined by both signal-dependent biosynthesis and physical stability of the pigments.

Collectively, mechanical damage converges on the MBW complex via two principal modules—JA-dependent derepression (exemplified by poplar) and WRKY-mediated feed-forward amplification (exemplified by grape)—whereas vibration-induced fading represents a post-transcriptional physical effect independent of regulatory signaling (**Table 9**).

**Table 9:** Mechanistic modules of mechanical-damage-induced anthocyanin regulation in woody ornamentals.

Representative species	Mechanistic module	Key regulators	MBW relationship	Functional logic
Poplar	JA‐dependent (repressor release)	*PtrJAZ1* degradation	Releases *PdMYB118*‐*PdTT8* complex	JA signal relieves repression, allowing MBW activation
Grape	WRKY‐JA feedforward (dual)	*VvWRKY5*	Directly binds *VvUFGT* (MBW target) + enhances JA synthesis	Simultaneous direct activation and signal amplification
Fruits (general)	Physical disruption (non-transcriptional)	–	MBW‐independent	Strong vibration disrupts pigment stability, causing fading

#### 4.7.2 Chemical stress

Chemical stressors such as pesticides (e.g., benzothiadiazole, BTH) and ozone can also induce anthocyanin accumulation, although the mechanistic understanding in woody perennials remains considerably less advanced than in herbaceous models. A provisional general model emerging from the limited evidence posits that chemical elicitors primarily trigger an intracellular oxidative burst, along with SA and ROS signaling cascades. This redox imbalance is perceived by yet-unidentified sensor proteins, which may converge on the MBW complex or directly upregulate structural genes—a functional mimic of the early signaling events in pathogen defense. Unlike mechanical or nutrient signals, chemical stress often bypasses classical hormone receptors and instead engages redox-sensitive transcription factors, though the exact molecular identity of these factors in woody tissues remains largely unknown.

The most compelling evidence comes from grapevine, where pre-harvest multiple sprays of BTH—a functional SA analog—significantly upregulate key structural genes in the flavonoid pathway, including *STS* and *UFGT*, leading to increased resveratrol and total anthocyanin content in berry skins (Iriti et al., 2004). This observation strongly suggests that SA-mediated signaling can activate the anthocyanin biosynthetic machinery, likely through the upregulation of an unknown MYB activator within the MBW complex; however, the intervening transcription factor has not yet been characterized in woody species under this specific chemical context. For ozone, phenotypic evidence of anthocyanin accumulation has been documented in several woody ornamentals, including *Acer truncatum*, in which O_3_ fumigation increases early-leaf anthocyanin content by 34.1%, *Prunus avium*/*Viburnum lantana*, which exhibit leaf reddening; and *Psidium guajava*, where anthocyanin content is significantly elevated after O_3_ exposure (Gravano et al., 2004; de Rezende and Furlan, 2009; Sun et al., 2024). In *A. truncatum*, ozone exposure has been shown to upregulate anthocyanin structural genes such as *CHS* and *DFR*, suggesting transcriptional activation of the pathway. However, a complete regulatory cascade—from O_3_ perception through upstream transcription factors to MBW complex activation—remains to be established in woody ornamentals; the upstream MYB or NAC regulators linking ozone-generated ROS signals to anthocyanin biosynthesis have not yet been identified in any woody species. For heavy metals, direct molecular evidence is even scarcer, with no confirmed regulatory cascade reported in ornamental trees or shrubs.

It should also be noted that the practical difficulties associated with chemical stress research in woody ornamentals—such as uneven agrochemical deposition due to tall/dense canopies and the technical bottleneck associated with stable transformation in heterozygous backgrounds—have historically constrained mechanistic progress. Beyond these operational issues, future research should prioritize: (i) identifying the primary redox sensors or SA receptors that perceive chemical elicitors in woody tissues; (ii) testing whether BTH/ROS signals converge on specific MYB members of the MBW complex using transient agroinfiltration assays; and (iii) developing woody-compatible VIGS systems to bypass the requirement for stable transformants.

In summary, chemical stress research in woody ornamentals currently provides the only well-characterized SA-mediated example so far, in the BTH-grape system, whereas molecular cascades for heavy metals and ozone remain entirely unresolved; these knowledge gaps define clear priorities for future validation using transient expression and VIGS approaches.

#### 4.7.3 Nutrient imbalances

Nutrient imbalance—particularly nitrogen (N) deficiency—is a potent trigger of anthocyanin accumulation in woody ornamentals, a response that can be consistent with the carbon-nitrogen (C/N) balance model. The general working model is as follows: under N-deficient conditions, plant primary growth is restrained, leading to a relative surplus of photosynthates (carbon skeletons). This metabolic shift activates a specific set of N-starvation-responsive MYB transcription factors, which in turn recruit bHLH and WD40 partners to assemble the MBW complex and drive the expression of anthocyanin structural genes. In this framework, anthocyanins act as a metabolic sink, diverting excess carbon skeletons into secondary metabolism while providing antioxidant protection against secondary oxidative stress induced by N limitation. Importantly, this N-specific pathway is not simply a general response to mineral deficiency; phosphorus (P) or potassium (K) deficiency does not reliably induce anthocyanins in most woody species, underscoring the unique signaling role of N status.

Empirical support for this model is best exemplified in eucalyptus (*Eucalyptus* spp.), where N deficiency specifically upregulates genes encoding MYB, bHLH, and WD40 components, followed by activation of structural genes and consequent purple anthocyanin accumulation in leaves and stems. Notably, supplementation with P or K fails to reverse this purple phenotype, indicating that N is the key nutritional determinant in this species (Zeng et al., 2024). While sporadic phenotypic observations of N-limited reddening exist in other taxa such as maple and poplar, the underlying molecular regulators have not been characterized in these species; therefore, eucalyptus currently stands as the only woody ornamental model with a fully delineated C/N-MYB regulatory axis. The regulation of anthocyanins by mineral elements is generally indirect—operating through widespread metabolic reprogramming rather than direct signal transduction—which makes mechanistic studies inherently more complex than those for light or temperature. The current priority is to identify the N-sensing components (e.g., nitrate transporters or *NLP* transcription factors) that link external N availability to MYB activation in woody species.

In summary, N deficiency operates through a C/N rebalancing mechanism coupled to N-specific MYB activation of the MBW complex—a model currently validated only in eucalyptus—whereas P/K deficiency lacks consistent molecular evidence; future work should prioritize identification of upstream N-sensing components in woody tissues.

## 5 Dynamic accumulation of anthocyanins under multiple environmental stresses

In natural or agricultural ecosystems, woody ornamental plants are frequently challenged by combinations of abiotic stresses, such as drought coupled with high temperature or low temperature combined with salinity. Unlike the linear responses to single stresses, anthocyanin accumulation under combined stresses emerges from the integration of multiple signaling networks, yielding outcomes that are not simply additive. These interactions can be categorized into three archetypes: synergistic, where dual stress signals converge on common nodes to produce supra-additive (> additive) transcriptional activation; antagonistic, where the positive effect of one signal is counteracted by negative regulation triggered by the other; and dominance, where one stress signal overrides the other, and the final phenotype is governed by the dominant factor (**Figure 5**). Guided by this conceptual framework, we dissect the integrative mechanisms of anthocyanin metabolic reprogramming under combined stresses in woody ornamentals at three layers: transcriptional regulation, hormonal crosstalk with metabolic trade-offs, and epigenetic accessibility.

**Figure 5:**
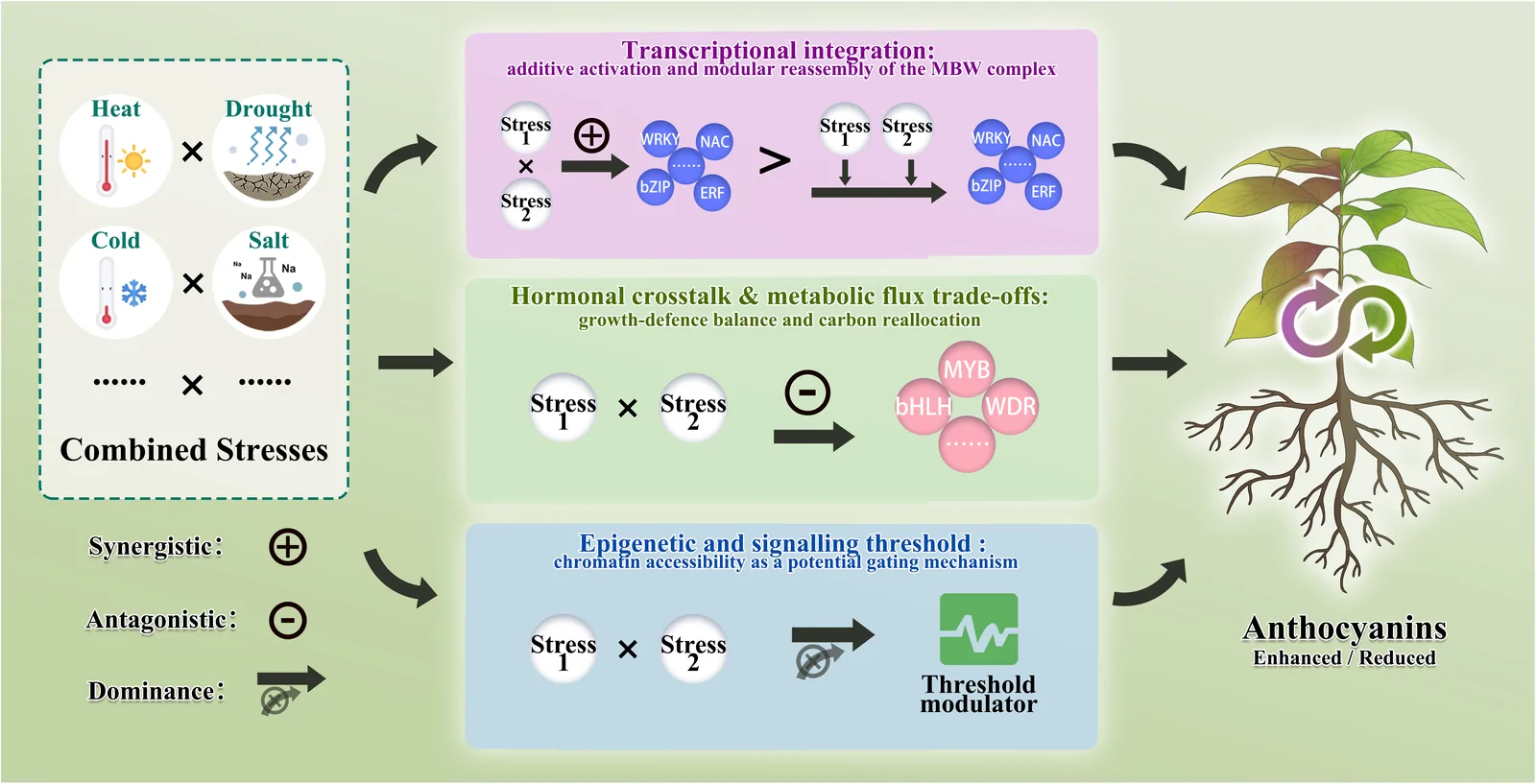
Integrative framework for anthocyanin accumulation under combined stresses.

(i) Transcriptional integration: supra-additive activation and modular reassembly of the MBW complex. The most direct integration occurs at the transcriptional level. When two stresses simultaneously activate the same upstream transcription factors, synergistic amplification can arise. A paradigmatic example is kiwifruit (*Actinidia chinensis*), where the combination of low temperature and light does not simply induce the anthocyanin pathway independently; instead, it synergistically promotes the formation of the *AcMYB10-AcbHLH42* heterodimer, which exhibits markedly enhanced binding affinity to the promoters of the structural genes *AcLDOX* and *AcF3GT*, leading to transcriptional outputs far exceeding the sum of individual stresses (Yu et al., 2019). This indicates that synergy at the molecular level is manifested as enhanced assembly efficiency of transcriptional complexes, rather than mere upregulation of individual genes.

(ii) Hormonal crosstalk and metabolic flux trade-offs: growth-defence balance and carbon reallocation. When divergent stresses trigger antagonistic hormonal signals, the output of the MBW pathway is constrained by metabolic competition or hormonal weighting. In apple, combined low-temperature and salt stress re-establishes gibberellin (GA) and abscisic acid (ABA) homeostasis via *MdGA2ox7*; the induced anthocyanins not only act as ROS scavengers but also serve as membrane stabilizers, producing a cross-protection effect (Yan et al., 2024a). However, this synergism is not universal. Studies on jujube (*Ziziphus jujuba*) fruit reveal an antagonistic mechanism involving metabolic flux allocation: elevated temperature (+1.5–2.5 °C) alone promotes soluble sugar accumulation, providing precursors for anthocyanin aglycone synthesis; however, when combined with drought (30–50% field capacity), drought dominates by activating ABA signaling, which redirects photosynthetic carbon flux primarily toward osmotic adjustment rather than the phenylpropanoid pathway, thereby suppressing pigment biosynthesis through substrate competition (Jiang et al., 2020a, 2020b). This underscores that the relative intensity and timing of upstream hormonal signals determine whether the MBW downstream pathway is empowered or deprived of carbon sources.

(iii) Epigenetic and signaling threshold layer: chromatin accessibility as a potential gating mechanism. Compared with transcriptional and hormonal layers, epigenetic modifications in woody plants under combined stresses remain largely unexplored; nevertheless, clues from single-stress studies suggest their potential as “integration gates”. For instance, under low-temperature alone, apple *MdCHS* and *MdUFGT* promoters undergo DNA demethylation, thereby opening chromatin for MBW complex binding (Yu et al., 2022). It is reasonable to postulate that under combined stresses (e.g., low temperature and salt), the two signals might operate via additive demethylation or cooperative rearrangement of histone marks (H3K9ac/H3K27me3) to lower the activation threshold of MBW target genes, and amplifying synergistic outputs. Although this hypothesis awaits experimental validation, it points to a critical future direction. While studies on herbaceous plants (e.g., UV-A-induced disease resistance in tomato, insect-feeding-enhanced drought tolerance in maize) offer references for hormonal crosstalk (Park and Ryu, 2016; González-García et al., 2023), woody perennials—given their perennial architecture and seasonal carbon dynamics—exhibit greater spatiotemporal complexity in combined-stress integration, calling for dedicated research frameworks.

To visually summarize these mechanistic differences, we compile representative cases of combined-stress studies in woody ornamental plants in **Table 10**. Collectively, anthocyanin accumulation under combined stresses depends on whether the MBW complex is synergistically hyperactivated at the transcriptional level or encounters antagonistic carbon deprivation at the hormone–metabolism interface, with epigenetic accessibility potentially serving as a “modulator” for threshold modulation. Future efforts should integrate time-resolved multi-omics (transcriptome–metabolome–chromatin accessibility) and *in vivo* functional validation in woody tissues (e.g., VIGS-based transient silencing) to systematically dissect the dynamic weighting of these integrative modules across diverse stress combinations and temporal sequences, thereby providing predictable molecular targets for combined-stress tolerance breeding.

**Table 10:** Representative cases and interaction mechanisms of anthocyanin regulation under combined stresses in woody ornamental plants.

Representative species	Combined stress combination	Interaction type	Key regulatory node/gene	Mechanism layer	Functional outcome
Apple (*Malus*)	Low temperature + Salinity	Synergistic/Cross‐protection	*MdGA2ox7* → anthocyanin accumulation	Hormonal crosstalk (GA/ABA rebalancing)	ROS scavenging, membrane stabilisation, enhanced dual tolerance
Jujube (*Ziziphus jujuba*)	High temperature + Drought	Antagonistic (drought‐dominant)	Carbon metabolic flux reprogramming (sugars vs. phenylpropanoids)	Metabolic trade‐off (substrate competition)	Pigment synthesis repressed; osmotic protection prioritized
Kiwifruit (*Actinidia chinensis*)	Low temperature + Light	Synergistic (supra‐additive)	*AcMYB10‐AcbHLH42* → *AcLDOX/F3GT*	Transcriptional complex hyperactivation (MBW)	Anthocyanin burst, photoprotection coupled with cold tolerance

## 6 Metabolic trade-offs under stress: defense investment versus growth cost

The preceding chapters have systematically elucidated the regulatory networks of anthocyanin biosynthesis under various stresses. However, a central question remains to be independently dissected: is stress-induced anthocyanin accumulation (or suppression) a passive consequence of stress damage, or an active resource reallocation strategy executed by plants? This question strikes at the heart of understanding plant survival strategies.

Accumulating evidence supports the active strategy as a general principle. Under drought and low temperature, anthocyanin synthesis represents “active investment”—in apple, the *MdVQ8*-*MdWRKY75* module actively stabilizes *MdMYB1* protein under salt stress to enhance MBW output (Gao et al., 2026); in *Prunus mume*, the *PmCBF03*-*PmMYB10* pathway is directly activated under low temperature (Meng et al., 2025)—all depending on the sequential initiation of specific signaling cascades rather than stress-induced metabolic disruption. Under high temperature, plants downregulate anthocyanin biosynthesis as a resource-conserving strategy: *MdGATA15* actively suppresses *MdMYB11* and *MdANS* in apple (Fang et al., 2025); *VvMYB44–1* directly represses *VvDFR* and *VvUFGT* in grape (Leng et al., 2025); and *PpHsfB2a* achieves reversible inhibition via ubiquitination-level changes in pear (Wang et al., 2025d). Even within the same stress type, the regulatory output is modulated in a stress-dose-dependent manner—overexpression of the blue-light photoreceptor *PagCRY1a/1b* in poplar causes massive anthocyanin accumulation accompanied by severe growth retardation and enhanced secondary xylem development, whereas *PagCRY1a/1b* knockout promotes vegetative growth (Chen et al., 2024b).

A regulatory logic that prioritizes stress tolerance while minimizing growth costs can be distilled into three mechanistic layers. (i) Carbon skeleton competition: anthocyanins share the phenylpropanoid pathway with core metabolites such as lignin; over-investment in secondary metabolism inevitably compromises primary growth—*PtrCRY1a/1b* overexpression in poplar leads to massive anthocyanin accumulation accompanied alongside severe dwarfing (Chen et al., 2024b). (ii) Hormonal antagonism: ABA/ET induced by low temperature and drought compete with IAA/BR induced by high temperature at the MBW node, with the output direction determined by relative signal strength. (iii) Energy dissipation cost: anthocyanin synthesis is an ATP- and NADPH-intensive process; the cost of the “red-leaf strategy” is manifested not only in carbon and potassium accumulation but also in inhibited nitrogen and phosphorus remobilization. Thus, the activity of this pathway is dynamically gated by stress cues, functioning as a resource-allocation rheostat that balances defense costs against growth demands—essentially an “investment algorithm” that seeks the optimal survival solution under finite resource constraints.

For breeding, this framework suggests moving beyond constitutive high-expression strategies toward: (i) editing negative regulators (e.g., *MdGATA15*, *VvMYB44-1*) to release the high-temperature brake, and (ii) employing stress-inducible promoters to drive synthesis genes, achieving stress-induced pigmentation without compromising growth under favorable conditions. Translating trade-offs from physiological concepts to actionable molecular targets will be key to climate-resilient breeding.

## 7 Conclusions and future directions

This review systematically synthesizes the regulatory frameworks and functional roles of anthocyanin metabolism in woody ornamental plants under diverse environmental stresses. A unifying theme emerges: although upstream stress perception and hormonal signals are species-divergent, the MBW (MYB-bHLH-WD40) transcriptional complex is highly conserved as the core execution hub in woody taxa. Under abiotic stresses, anthocyanin outputs are stress-specific—drought and cold generally induce accumulation, high temperatures suppress it, and heavy metals uniquely trigger chelation-dependent detoxification. Under biotic stresses, anthocyanins act as chemical barriers and visual deterrents, coordinated by hormonal (SA/JA/ethylene) and transcriptional (*NPR1-TGA*, *WRKY*) cascades. These regulatory events are not isolated; rather, they operate through integrated functional modules—transcriptional reprogramming, epigenetic modifications, hormonal crosstalk, and vacuolar sequestration—that together enable fine-tuned acclimation. Critically, the perennial habit, seasonal stress memory, and complex canopy architecture of woody plants set their anthocyanin regulatory logic apart from that of herbaceous model systems spatially and temporally.

Despite substantial progress, three major bottlenecks remain. First, causality is largely lacking—most evidence relies on heterologous overexpression and phenotypic correlations; direct loss-/gain-of-function assays in woody tissues via VIGS or CRISPR are scarce, leaving the *in vivo* necessity of anthocyanins for stress tolerance formally unproven. Second, the regulatory logic under combined stresses (e.g., drought and heat, cold and salt) remains obscure, as most studies focus on single stresses; the synergistic, antagonistic, or dominant integration of multiple signals—and their impact on metabolic flux redistribution—is only beginning to be explored. Third, the upstream sensor nodes (e.g., osmosensors, metal ion receptors) and the complete cascade involving epigenomic remodeling (DNA methylation, histone modifications, non-coding RNAs) and post-translational regulation are almost entirely uncharacterized in woody ornamentals, severely limiting the predictive power of current models.

Addressing these gaps will directly enable translational applications for stress resilience breeding and genetic engineering. At the breeding level, GWAS and population genomics can harness natural allelic variation, coupled with marker-assisted selection, to identify germplasm with enhanced anthocyanin content and broad-spectrum stress tolerance. At the engineering level, CRISPR/Cas9-mediated editing of upstream MBW repressors or stress-inducible overexpression of key MYB activators offers a sustainable route to enhance anthocyanin-mediated protection without compromising growth or ornamental quality. Long-term, integrating high-throughput phenotyping with field-based environmental monitoring will be essential to validate laboratory discoveries under fluctuating real-world conditions, ultimately positioning anthocyanin metabolism as an engineerable hub for climate-smart forestry and horticulture.
