# Supplementary material for: Anthocyanins in woody ornamental plants: biosynthesis, regulatory networks, and trade-offs in stress adaptation
Source: Front Plant Sci. 2026 Sep 28;17:1966821. doi: 10.3389/fpls.2026.1966821 (PMC13655925; doi:10.3389/fpls.2026.1966821)
Supplement: Supplementary file 1 [file Table1.docx]

Table S1. The roles of genes involved in anthocyanin biosynthesis and plant responses to diverse biotic and abiotic stresses.

| Gene Name | Regulatory category | Changes in anthocyanin content | Plant Species | Family | Environmental Factors | References |
| --- | --- | --- | --- | --- | --- | --- |
| WRKY11 | Transcriptional regulation (WRKY) | Up | Pear (Pyrus communis ‘Starkrimson’) | Rosaceae | UV-B (binds PcPIF3 promoter) | Cao et al., 2026 |
| PIF3 | Light signaling (PIF) | Up | Pear (Pyrus communis ‘Starkrimson’) | Rosaceae | UV-B (activates PcMYB10) | Cao et al., 2026 |
| UGT79B1 | Anthocyanin glycosyltransferase | Up | Blood orange (Citrus sinensis) | Rutaceae | UV-B | Yang et al., 2025 |
| MYB10 | Transcriptional regulation (MYB) | Up | Pear (Pyrus communis ‘Starkrimson’) | Rosaceae | UV-B | Cao et al., 2026 |
| EBF1 | Ubiquitination (E3 ligase) | Down (via MdVQ8 degradation) | Apple (Malus domestica) | Rosaceae | Salt stress (promotes MdVQ8 degradation) | Gao et al., 2026 |
| VQ8 | Transcriptional co-regulator (VQ motif) | Up | Apple (Malus domestica) | Rosaceae | Salt stress (ethylene and calcium synergy) | Gao et al., 2026 |
| MYB176 | Transcriptional regulation (MYB) | Up | Areca palm (Areca catechu) | Arecaceae | Salt stress (activates AcCHS5) | Jiang et al., 2025 |
| CHS5 | Anthocyanin structural gene (CHS) | Up | Areca palm (Areca catechu) | Arecaceae | Salt stress | Jiang et al., 2025 |
| WRKY75 | Transcriptional regulation (WRKY) | Up | Apple (Malus domestica) | Rosaceae | Salt stress | Gao et al., 2026 |
| MYB1 | Transcriptional regulation (MYB) | Up | Apple (Malus domestica) | Rosaceae | Salt stress | Gao et al., 2026 |
| TCP46 | Transcriptional regulation (TCP) | Up | Apple (Malus domestica) | Rosaceae | Salt stress | Gao et al., 2026 |
| CML15 | Calcium signaling (calmodulin-like) | Up | Apple (Malus domestica) | Rosaceae | Salt stress | Gao et al., 2026 |
| DFR, ANS, FLS | Structural genes | Up | Grape | Vitaceae | Salt stress | Chen et al., 2025d |
| 3GT2 | Anthocyanin modification (glycosyltransferase) | Up | Rhododendron (Rhododendron agastum) | Ericaceae | Salt and drought (overexpression enhances tolerance) | Luo et al., 2025 |
| PP2C9 | Hormonal signaling (ABA, negative regulator) | Up (due to repression of negative regulator) | Grape (Vitis vinifera) | Vitaceae | Red/blue light (downregulated) | Kondo et al., 2025 |
| SnRK2.6 | Hormonal signaling (ABA, positive) | Up | Grape (Vitis vinifera) | Vitaceae | Red/blue light (activates VvABF2) | Kondo et al., 2025 |
| ABF2 | Hormonal signaling (ABA, bZIP) | Up | Grape (Vitis vinifera) | Vitaceae | Red/blue light | Kondo et al., 2025 |
| MYBa1 | Transcriptional regulation (MYB) | Up | Grape (Vitis vinifera) | Vitaceae | Red/blue light | Kondo et al., 2025 |
| UFGT | Anthocyanin structural gene (UFGT) | Up | Grape (Vitis vinifera) | Vitaceae | Red/blue light | Kondo et al., 2025 |
| JAZ1 | Jasmonate signaling (repressor) | Up (via release of activation complex) | Poplar (Populus trichocarpa) | Salicaceae | Mechanical wounding (degraded by JA) | Wang et al., 2020 |
| WRKY5 | Transcriptional regulation (WRKY) | Up | Grape (Vitis vinifera) | Vitaceae | Mechanical wounding | Zhang et al., 2024d |
| MYBA1 | Transcriptional regulation (MYB) | Up | Grape (Vitis vinifera) | Vitaceae | Mechanical wounding | Zhang et al., 2024d |
| LOX | Jasmonic acid biosynthesis (lipoxygenase) | Up (via JA) | Grape (Vitis vinifera) | Vitaceae | Mechanical wounding | Zhang et al., 2024d |
| MYB118 | Transcriptional regulation (MYB) | Up | Poplar (Populus trichocarpa) | Salicaceae | Mechanical wounding | Wang et al., 2020 |
| TT8 | Transcriptional regulation (bHLH) | Up | Poplar (Populus trichocarpa) | Salicaceae | Mechanical wounding | Wang et al., 2020 |
| LDOX | Anthocyanin structural gene (LDOX) | Up | Kiwifruit (Actinidia chinensis) | Actinidiaceae | Low temperature + light (combined stress) | Yu et al., 2019 |
| F3GT | Anthocyanin structural gene (UFGT) | Up | Kiwifruit (Actinidia chinensis) | Actinidiaceae | Low temperature + light | Yu et al., 2019 |
| MYB10 | Transcriptional regulation (MYB) | Up | Kiwifruit (Actinidia chinensis) | Actinidiaceae | Low temperature + light | Yu et al., 2019 |
| bHLH42 | Transcriptional regulation (bHLH) | Up | Kiwifruit (Actinidia chinensis) | Actinidiaceae | Low temperature + light | Yu et al., 2019 |
| YABBY2 | Transcriptional regulation (YABBY) | Up | Apple (Malus ‘Pinkspire’) | Rosaceae | Low temperature (promotes ABA synthesis) | Chen et al., 2025b |
| CHS2 | Anthocyanin structural gene (CHS) | Up | Paper mulberry (Broussonetia papyrifera) | Moraceae | Low temperature (overexpression promotes cold tolerance) | Fu et al., 2025 |
| COP1 | Ubiquitination (E3 ligase, repressor) | Down | Red maple (Acer rubrum) | Sapindaceae | Low temperature (mediates ArMYB89 degradation) | Shah et al., 2024 |
| NAC42 | Transcriptional regulation (NAC) | Up | Pear (Pyrus spp.) | Rosaceae | Low temperature (interacts with PyMYB10) | Zhang et al., 2024b |
| ETR2 | Ethylene signaling (receptor) | Up | Paper mulberry (Broussonetia papyrifera) | Moraceae | Low temperature (induces sugar synthesis) | Fu et al., 2025 |
| MYB11 | Transcriptional regulation (MYB) | Up | Apple (Malus ‘Pinkspire’) | Rosaceae | Low temperature (forms MBW with MpbHLH79) | Chen et al., 2025b |
| GST | Anthocyanin transport (GST) | Up | Paper mulberry (Broussonetia papyrifera) | Moraceae | Low temperature (enhances transport) | Fu et al., 2025 |
| MYB89 | Transcriptional regulation (MYB) | Up | Red maple (Acer rubrum) | Sapindaceae | Low temperature (activates ArUGT52) | Shah et al., 2024 |
| AREB1 | Transcriptional regulation (bZIP) | Up | Tomato (Solanum lycopersicum) | Solanaceae | Low temperature (ABA-dependent) | Xu et al., 2023b |
| PAL, CHS, DFR, UFGT | Structural genes | Up | Blood orange | Rutaceae | Low temperature (8°C) | Chen et al., 2025a |
| MYB16 | Transcriptional regulation (MYB) | Up | Acer pseudosieboldianum | Sapindaceae | Low temperature (52-fold upregulation) | Zhang et al., 2025 |
| MYB31 | Transcriptional regulation (MYB) | Up | Acer pseudosieboldianum | Sapindaceae | Low temperature (28-fold upregulation) | Zhang et al., 2025 |
| UGT52 | Anthocyanin glycosyltransferase | Up | Red maple (Acer rubrum) | Sapindaceae | Low temperature | Shah et al., 2024 |
| bHLH79 | Transcriptional regulation (bHLH) | Up | Apple (Malus ‘Pinkspire’) | Rosaceae | Low temperature | Chen et al., 2025b |
| MYB10 | Transcriptional regulation (MYB) | Up | Pear (Pyrus spp.) | Rosaceae | Low temperature | Zhang et al., 2024b |
| DREB1E | Transcriptional regulation (DREB) | Up | Sweet cherry (Prunus avium) | Rosaceae | Light and gibberellin | Xiao et al., 2025 |
| D8 | (unknown, likely signaling) | Up | Sweet cherry (Prunus avium) | Rosaceae | Light and gibberellin | Xiao et al., 2025 |
| HY5 | Light signaling (HY5) | Up | Sweet cherry (Prunus avium) | Rosaceae | Light and gibberellin | Xiao et al., 2025 |
| ERF109 | Transcriptional regulation (ERF) | Up | Apple (Malus domestica) | Rosaceae | Light (after bag removal) | Ma et al., 2021 |
| HsfB2a | Transcriptional regulation (HSF) | Down | Pear (Pyrus pyrifolia) | Rosaceae | High temperature (protein accumulation, inhibits light signaling) | Wang et al., 2025b |
| BZR1 | Transcriptional regulation (BZR) | Down | Apple (Malus domestica) | Rosaceae | High temperature (positive feedback with MdLBD37, represses) | Li et al., 2025a |
| BBX32 | Transcriptional regulation (BBX) | Up (peel) | Peach (Prunus persica) | Rosaceae | High temperature (peel-specific) | Huang et al., 2024 |
| ARF3 | Hormonal signaling (auxin) | Down | Grape (Vitis vinifera) | Vitaceae | High temperature (interacts with VvFHY3, triggers ER stress) | Sun et al., 2025 |
| ZAT5 | Transcriptional regulation (ZAT) | Up (flesh) | Peach (Prunus persica) | Rosaceae | High temperature (flesh-specific) | Huang et al., 2024 |
| GATA15 | Transcriptional regulation (GATA, repressor) | Down | Apple (Malus domestica) | Rosaceae | High temperature (directly represses MdMYB11 and MdANS) | Fang et al., 2025 |
| BBX32 | Transcriptional regulation (BBX) | Up | Grape (Vitis vinifera) | Vitaceae | High temperature (activates VvBZR1, promotes anthocyanin) | Qiu et al., 2025 |
| MYB308 | Transcriptional regulation (MYB, repressor) | Down | Apple (Malus domestica) | Rosaceae | High temperature (activated by MdGATA15) | Fang et al., 2025 |
| LBD37 | Transcriptional regulation (LBD, repressor) | Down | Apple (Malus domestica) | Rosaceae | High temperature | Li et al., 2025a |
| FHY3 | Light signaling (FAR-RED ELONGATED HYPOCOTYL3) | Down | Grape (Vitis vinifera) | Vitaceae | High temperature | Sun et al., 2025 |
| BZR1 | Transcriptional regulation (BZR) | Up | Grape (Vitis vinifera) | Vitaceae | High temperature | Qiu et al., 2025 |
| UVR8 | Photoreceptor (UV-B receptor) | Up (via CpMYB12) | Wintersweet (Chimonanthus praecox) | Calycanthaceae | High light / UV-B | Wang et al., 2025d |
| MYB12 | Transcriptional regulation (MYB, activator) | Up | Wintersweet (Chimonanthus praecox) | Calycanthaceae | High light / UV-B | Wang et al., 2025d |
| MYB4 | Transcriptional regulation (MYB, repressor) | Down (antagonizes CpMYB12) | Wintersweet (Chimonanthus praecox) | Calycanthaceae | High light / UV-B | Wang et al., 2025d |
| C4H, LAR, ANS | Structural genes | Up | Tea plant (Camellia sinensis) | Theaceae | Heavy metal (Lead) + Melatonin | Li et al., 2025b |
| NPR1‑TGA2.2 | Hormonal signaling (SA) + Transcriptional | Up | Apple (Malus domestica) | Rosaceae | Fungal infection (Valsa mali, Botryosphaeria dothidea) | Li et al., 2024 |
| miR156b | Non-coding RNA (post-transcriptional) | Up | Grape (Vitis vinifera) | Vitaceae | Drought (via ABA signaling, targets *VvSBP8/13*) | Guo et al., 2024 |
| miR172d | Non-coding RNA | Up | Tree peony (Paeonia ostii) | Paeoniaceae | Drought (repressed, leading to PoARR induction) | Li et al., 2026 |
| ARR | Transcriptional (response regulator) | Up | Tree peony (Paeonia ostii) | Paeoniaceae | Drought (activated, enhances antioxidant system) | Li et al., 2026 |
| ABI5 | Hormonal signaling (ABA) + Transcriptional | Up | Jujube (Ziziphus jujuba) | Rhamnaceae | Drought (ABA and ethylene synergy) | Wang et al., 2025a |
| EIN3 | Hormonal signaling (Ethylene) + Transcriptional | Up | Jujube (Ziziphus jujuba) | Rhamnaceae | Drought (ABA and ethylene synergy) | Wang et al., 2025a |
| bHLH3 | Hormonal signaling (Ethylene) + Transcriptional | Up | Crabapple (Malus spectabilis) | Rosaceae | Drought | Xu et al., 2025b |
| F3'H | Hormonal signaling (Ethylene) + Transcriptional | Up | Crabapple (Malus spectabilis) | Rosaceae | Drought | Xu et al., 2025b |
| ERF17 | Hormonal signaling (Ethylene) + Transcriptional | Up | Crabapple (Malus spectabilis) | Rosaceae | Drought | Xu et al., 2025b |
| CaM1‑MYB108 | Hormonal signaling (Ca²⁺) + Transcriptional cascade | Up | Jujube (Ziziphus jujuba) | Rhamnaceae | Drought | Wang et al., 2025a |
| MYB75, GSTFL11 | Drought stress (transcriptional activation and transport) | Up | Rose (Rosa chinensis) | Rosaceae | Drought | Ma et al., 2025 |
| GA2ox7 | Hormonal/developmental (GA oxidase) | Up | Apple (Malus domestica) | Rosaceae | Cold and salt (cooperates with melatonin) | Yan et al., 2024b |
| TT12 | Anthocyanin transport (MATE transporter) | Up | Lycium ruthenicum | Solanaceae | Cadmium stress (sequesters Cd-anthocyanin complexes) | Ai et al., 2025 |
| AN2 | Transcriptional regulation (MYB, activator) | Up | Lycium ruthenicum | Solanaceae | Cadmium stress (promotes anthocyanin & carotenoids) | Ai et al., 2025 |
| BBX24 | Transcriptional regulation (BBX) | Up | Mango (Mangifera indica) | Anacardiaceae | Blue light (activates MiMYB1) | Pan et al., 2025 |
| BBX27 | Transcriptional regulation (BBX) | Up | Mango (Mangifera indica) | Anacardiaceae | Blue light | Pan et al., 2025 |
| MYB1 | Transcriptional regulation (MYB) | Up | Mango (Mangifera indica) | Anacardiaceae | Blue light | Pan et al., 2025 |
